# Moderate Signal Enhancement in Electrospray Ionization Mass Spectrometry by Focusing Electrospray Plume with a Dielectric Layer around the Mass Spectrometer’s Orifice

**DOI:** 10.3390/molecules29020316

**Published:** 2024-01-08

**Authors:** Zi Qing Chua, Gurpur Rakesh D. Prabhu, Yi-Wun Wang, Chamarthi Maheswar Raju, Krzysztof Buchowiecki, Ochir Ochirov, Decibel P. Elpa, Pawel L. Urban

**Affiliations:** 1 Department of Chemistry, National Tsing Hua University, 101, Section 2, Kuang-Fu Rd., Hsinchu 300044, Taiwanho258077@gmail.com (Y.-W.W.); maheswarrajujob@gmail.com (C.M.R.); chris.buchowiecki@gmail.com (K.B.); ochirochirochir@gmail.com (O.O.); elpa.decibel@gmail.com (D.P.E.); 2 Frontier Research Center on Fundamental and Applied Sciences of Matters, National Tsing Hua University, 101, Section 2, Kuang-Fu Rd., Hsinchu 300044, Taiwan

**Keywords:** electrospray ionization, ion source, mass spectrometry, microdroplets, sensitivity

## Abstract

Electrospray ionization (ESI) is among the commonly used atmospheric pressure ionization techniques in mass spectrometry (MS). One of the drawbacks of ESI is the formation of divergent plumes composed of polydisperse microdroplets, which lead to low transmission efficiency. Here, we propose a new method to potentially improve the transmission efficiency of ESI, which does not require additional electrical components and complex interface modification. A dielectric plate—made of ceramic—was used in place of a regular metallic sampling cone. Due to the charge accumulation on the dielectric surface, the dielectric layer around the MS orifice distorts the electric field, focusing the charged electrospray cloud towards the MS inlet. The concept was first verified using charge measurement on the dielectric material surface and computational simulation; then, online experiments were carried out to demonstrate the potential of this method in MS applications. In the online experiment, signal enhancements were observed for dielectric plates with different geometries, distances of the electrospray needle axis from the MS inlet, and various compounds. For example, in the case of acetaminophen (15 μM), the signal enhancement was up to 1.82 times (plate B) using the default distance of the electrospray needle axis from the MS inlet (*d* = 1.5 mm) and 12.18 times (plate C) using a longer distance (*d* = 7 mm).

## 1. Introduction

Electrospray ionization (ESI)—which was first coupled with mass spectrometry (MS) in the 1980s by the research groups of John B. Fenn [1] and Lidija N. Gall [2,3] independently—has become one of the prominent atmospheric pressure ionization techniques for MS [4,5,6]. Infusing a liquid sample through a metal capillary while applying a potential difference between the capillary and a counter electrode produces charged droplets from the liquid surface when the electrostatic repulsive force is larger than the surface tension of the liquid [5]. Followed by continuous desolvation and Coulombic jet fissions, gas-phase ions are generated and then introduced into the mass spectrometer’s inlet [7,8]. Some distinct advantages include multiple charging and the possibility of analyzing thermolabile and non-volatile analytes without fragmentation. Facile coupling with online separation techniques has also contributed to the wide adoption of this technique [9]. Thus, ESI-MS is routinely applied in many research fields, including proteomics [10] and biomedicine [11].

Apart from many advantages, ESI also has some limitations. The sensitivity of ESI-MS is limited by ionization and ion transmission efficiencies [12,13]. Ionization efficiency refers to the amount of analyte present in the liquid phase that is converted to the gas-phase ions [13,14,15]. It depends on multiple factors, such as the natural properties of the analyte [15,16], solvent properties (surface tension, conductivity, and dielectric constant) [17], solvent composition [18,19], sample flow rate [20,21,22,23], and ESI-MS interface [13,24]. On the other hand, ion transmission efficiency is defined as the proportion of gas-phase analyte ions entering the mass spectrometer’s inlet [25]. The polydispersity of the ESI plume limits the number of ions that are successfully introduced into the vacuum region [26,27]. The repulsion of like-charged ions within the spray plume causes a rapid expansion of radial plume size during the propagation from the ESI emitter to the mass analyzer [28]. Thus, the dispersed area of electrospray ions is much larger than the MS inlet size. The transmission efficiency of ESI-generated ions may be well below 1% [29,30,31]. Although the transmission efficiency may be improved by enlarging the MS inlet, this method also requires a higher-performance vacuum pump to ensure the low-pressure environment within the mass analyzer [13,32,33,34].

Various attempts have been made to improve the performance of ESI. NanoESI—disclosed by Wilm and Mann in the 1990s—operates with the nL min^−1^ flow rates and, in some cases, uses emitters with diameters as small as 1–2 μm [26,35]. The improvement is attributed to the production of smaller droplets (nanodroplets), which have a higher charge-to-volume ratio. NanoESI boosts mass spectrometer sensitivity dramatically, providing an ionization efficiency of 100% in certain conditions [36,37]. Moreover, nanoESI also provides higher salt tolerance than conventional ESI [38]. The transmission efficiency of nanoESI is also high because the small plume allows more ions to reach the MS inlet. Another way to enhance the ionization of analytes in ESI is by using certain additives. The additives in the sample solvent increase sample conductivity, promote charging [39,40], and sometimes also improve chromatographic separation [41]. On the other hand, different ways have been tried to improve the transmission efficiency between the ESI source and mass analyzer [42]. One of the common ways to improve transmission is the adoption of an ion funnel [43,44]. By applying radio frequency and direct current voltages into stacked ring electrodes with gradually smaller inner diameters, the ions can be efficiently focused into smaller radial clouds with a high ion flux [45,46]. Further, some researchers attempted to focus ions by incorporating electrode components with specific geometry [47,48]. The size of the droplet plume could be decreased by applying voltages with the same polarity, leading to a smaller expansion of the plume [49]. In other work, Iyer et al. implemented 3D-printed electrodes of different shapes, which are able to manipulate ion beams in the open-air environment [48]. Moreover, multiplexing emitters in different arrangements can be used as a potential method to enhance MS signal intensities [50,51,52,53]. In particular, the combination of multiple emitters and ion funnel can improve sensitivity [54,55]. Additionally, ESI performance could be improved by sorting droplets using different physical conditions (e.g., inertia [28,56], sound [27]).

Apart from its applications in MS, electrospray has been used in other areas, for example, to pattern particles with the aid of a dielectric mask on the substrate surface [57,58,59]. Since deposited charges cannot dissipate to the ground electrode, they are accumulated on the dielectric surface [60,61]. As a result of the repulsive Coulomb force between charged particles of the same polarity, the incoming charged particles are confined in a conductive zone underneath [62]. Thus, we believe that this method can also be utilized in MS to confine charged droplets or ions present in the electrospray plume.

Here, we aimed to develop a simple and convenient method to focus the electrospray plume by placing a dielectric plate in front of the MS inlet. The rationale of this approach is that the electric field can be modified in front of the MS inlet due to charge accumulation on the dielectric surface. The relatively dense electric field—adjusted by the dielectric—may focus charged droplets and gas phase ions into the MS inlet. We have verified this method by conducting a series of offline and online experiments.

## 2. Experimental Section

### 2.1. Chemicals

Methanol (LC-MS grade) and water (for chromatography, LiChrosolv) were purchased from Merck (Darmstadt, Germany). Ethanol (anhydrous, 99.5+%) was purchased from Echo Chemical (Miaoli, Taiwan). Acetaminophen (meets USP testing specs., 98.0–102.0%), ammonium acetate (≥99%, for HPLC), fluorescein (for fluorescence, free acid), gly his, L-citrulline (≥98%, for TLC), L-glutathione (reduced, ≥98%), and L-lysine (≥98%, for TLC) were purchased from Sigma-Aldrich (St. Louis, MO, USA). Ammonium hydroxide (30–33%) was purchased from Honeywell (Charlotte, NC, USA). Angiotensin II (human recombinant) and cloxacillin sodium salt were purchased from Alfa Aesar (Ward Hill, MA, USA). Cytochrome *c* (90%, from horse heart muscle), formic acid (>98%), glycine (≥99%), L-alanine (99%), and L(-)-tryptophan (99%) were purchased from Acros Organics (Geel, Belgium). Ubiquitin (human recombinant) was purchased from R&D Systems (Minneapolis, MN, USA). Custom synthesized peptides with different lengths [HPF, HPFHPF, HPFHPFHPF (H: histidine; P: proline; F: phenylalanine)] were purchased from BioAb (Taipei, Taiwan).

For the offline experiment, the fluorescein stock solution (2.5 × 10^−3^ M) was prepared in 75% (*v*/*v*) aqueous methanol solution in advance. Subsequently, 300 µL of the stock solution and 20 µL ammonium hydroxide (30–33% NH_3_ in water) were pipetted to a volumetric flask. The mixture was finally diluted to the final volume of 10 mL with 75% (*v*/*v*) aqueous methanol solution to obtain a final fluorescein concentration of 7.5 × 10^−5^ M. For the online experiments, all of the chemicals were prepared in 50% (*v*/*v*) aqueous ethanol solution with a final analyte concentration of 15 μM unless noted otherwise.

### 2.2. Offline Setup

**Figure 1:**
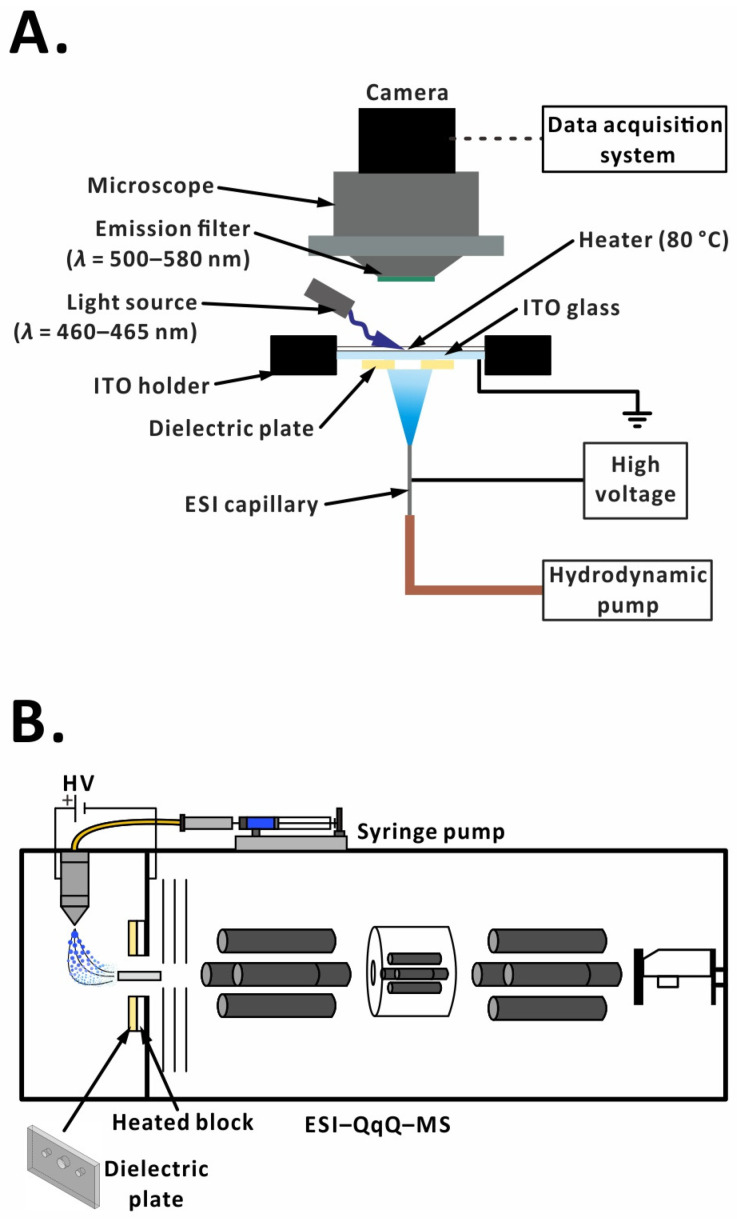
Simplified scheme of experimental setup: (**A**) offline experiment; (**B**) online experiment.

The offline setup consists of three parts: (i) a hydrodynamic-driven pump system, (ii) an ESI system, and (iii) a microscope-based data acquisition system (Figure 1A). Pressurized nitrogen gas (inlet pressure, 68.9 kPa) was used in the hydrodynamic pump. A digital manometer (AZ 8230; AZ Instrument Corporation, Taichung, Taiwan) was connected to the sample vial to measure the headspace pressure (Figure S1). During the injection, the headspace pressure was maintained at 8.5 kPa to ensure that the 7.5 × 10^−5^ M fluorescein in 75% (*v*/*v*) aqueous methanol solution was transferred at ~10 µL min^−1^ by fused silica capillary tubing (I.D. 150 µm, O.D. 375 µm, length 49.8 cm; GL Science, Tokyo, Japan). Electrospray occurred between a grounded indium tin oxide (ITO) glass (25 mm width × 75 mm length; thickness: 0.7 mm; part no. UR-ITO007-0.7MM; Flamegold Material, Taoyuan, Taiwan) and a stainless-steel capillary (I.D. 100 µm, O.D. 270 µm, length 82.5 mm; part no. 225-14948-91, Shimadzu, Kyoto, Japan), which was connected to a positive polarity power supply (potential: +4.5 kV; model no. MPS10P10/24/VCC; Spellman High Voltage Electronics Corporation, Hauppauge, NY, USA). The fluorescein droplets—deposited on the ITO surface—were illuminated with a blue LED (wavelength, 460–465 nm; diameter: 8 mm; applied voltage: 9 V; Centenary Materials, Hsinchu, Taiwan) located above the ITO glass holder. In order to prevent the accumulation of droplets on the ITO glass surface, the ITO glass was preheated to 80 °C for 10 min using a transparent heater (model no. NT22-1A; FlexSo Technology, Miaoli, Taiwan), attached to the surface before every analysis to improve the desolvation process. The distance between the ESI capillary tip and the grounded surface was maintained at 10 mm by an *XYZ* translation stage throughout the whole experiment. The customized dielectric plates with different orifice sizes (2.5 mm, 5 mm, 7.5 mm, 10 mm) were made of ceramic (99.7% aluminum oxide; material: AM997A; Ferrotec Holdings Corporation, Tokyo, Japan; Figures S2A and S3B). The dielectric plate was fixed closely under the ITO glass without any spacing using a 3D-printed custom-made holder made of acrylonitrile–butadienestyrene (printer: UP Plus, 3DP-14-4D; Beijing TierTime Technology, Beijing, China; Figure S4).

Excitation light and electrospray voltage were on all the time during the experiment. Before the experiment started, the capillary tip was maintained at the center of the orifice. A solenoid valve (nominal pressure: 0–6 bar; nominal voltage: 12 V; part no. 2W025-06; Thai Xin Machinery, Kaohsiung, Taiwan) was connected to one end of a steel union cross (tubing O.D. 3.2 mm; material: stainless steel; part no. SS-200-4; Swagelok, Solon, OH, USA), and acted as a switch for the sample supply. When the solenoid valve is closed, the sample solution in the vial is forced and flows into the ESI capillary. When the solenoid valve is opened, the gas flows out from the valve. The industrial complementary metal-oxide-semiconductor camera (model no. DFK 33UX174; The Imaging Source, Taipei, Taiwan) was fitted on a stereo microscope (model no. SMZ745T; Nikon, Tokyo, Japan) with a 33.5× magnification ratio (0.67× objective lens and 50× extension tube) to observe the locations of the deposited electrospray droplets. One layer of emission filter (no. 090, transmission wavelength: ∼500–580 nm, FWHM; maximum transmittance wavelength: 540 nm; Rosco, Stamford, CT, USA) was placed in front of the lens of the microscope to eliminate scattered excitation light. To obtain a series of clear fluorescence images, the Imaging Express software (version 1.1.0.23; The Imaging Source, Taipei, Taiwan) was used. The camera parameters were brightness, 0 dB; exposure time, 0.1 s; gain value, 25 dB. Images were taken for a duration of 10 s. For the first 3 s, images were captured without turning on the ESI. After 3 s, ESI was started by closing the solenoid valve.

### 2.3. Computational Simulation of Electric Field

The focusing effect due to dielectric material is caused mainly by an interplay of two forces—attracting force between charged particles and exposed grounded electrode and Coulombic repulsion from the charge buildup on dielectric material [63]. Due to the landing on dielectric material, the charged particles are unable to dissipate the charge, which leads to charge accumulation and alteration of the electric field. In order to verify the feasibility of focusing electrospray droplets by a perforated dielectric plate, we first performed a simulation of the electric field in the offline setup in the presence and absence of the dielectric plate using COMSOL Multiphysics software (version 6.1.0.357; COMSOL, Burlington, MA, USA). Details of the model used in the simulation are provided in Figure S5. The simulation includes three components: ESI capillary, grounded electrode, and dielectric plate. The boundary conditions are as follows: voltage applied to the ESI capillary, +4.5 kV; voltage of grounded plate, 0 V; relative permittivity of ESI capillary and grounded electrode, 1; relative permittivity of the dielectric plate, 5.7; space between the ESI capillary and the ground electrode was filled with the air at atmospheric pressure; relative permittivity of the air, 1.

### 2.4. Measurement of Surface Charge Density

To evaluate the surface charge density for simulation purposes, we measured the charge deposited on the dielectric surface in the offline experimental setup (Figure S6A). A house-built electrostatic probe was used. The detection area was made of copper plate with an area of 10 × 10 mm. The probe was connected to a trans-impedance amplifier (gain setting: low noise, 10^5^ V A^−1^; model no. DLPCA-200; FEMTO Messtechnik, Berlin, Germany) to amplify the signals. The amplified signals were acquired using a high-resolution data logger (single-ended; sample interval: 60 ms; sample mode: average; model no. ADC-20; Pico Technology, St Neots, UK) connected to a computer. Prior to every analysis, the distance between the ground electrode and the ESI capillary was fixed at 10 mm. A flat dielectric plate with 5 mm orifice was fixed using the holder. After that, baseline data points were obtained for 60 s. A voltage of 4.5 kV was applied to the ESI capillary in this experiment. Then, 75% (*v*/*v*) aqueous methanol solution was electrosprayed with a flow rate of ~10 µL min^−1^ for different time intervals (0–5 min). After the electrospray was turned off, the surface charge was detected by the probe for one minute. Time-dependent data were then recorded by the computer for further analysis. During data treatment, baseline subtraction was performed by subtracting the average of the blank values (0–60 s) from each data point. Subsequently, voltage (*V*) was converted into current (*I*), taking into account the gain factor (GF = 1 × 10^5^ V A^−1^):

(1) $$I=\frac{V}{GF}$$

Since current is defined as the rate of charge flowing through in a specific time, the value of the charge (*q*) can be computed using the equation [64]:

(2) $$q=\int_{t_{1}}^{t_{2}}I\;dt$$

where *t*_1_ and *t*_2_ are the start and end times of the measurement. The plots of current vs. time were obtained experimentally, and the area under the curve was determined using the peak integration function in OriginPro software (version 8.5; OriginLab, Northampton, MA, USA). After the peak integration, the surface charge (*Q*_surface_) was obtained by dividing the total charge (*q*) value by the surface area (*A*_p_) of the probe (1 × 10^−4^ m^2^):

(3) $$Q_{surface}=\frac{q}{A_{p}}\;$$

### 2.5. Offline Experiment Data Analysis

After every experiment, 60 consecutive images—including the background and electrosprayed droplets—were processed in the following procedure:

1. The raw image sequence was imported into ImageJ software (version 1.53k; National Institutes of Health, Bethesda, MD, USA; see the Supporting Information for the code to execute points 1–4).
2. The frameset was subjected to background subtraction. The first frame was subtracted from all the consecutive images.
3. A median filter (radius: 2 pixels) was applied to all the images to lower the noise. Then, the images were converted to 8-bit mode.
4. The processed images were transformed into binary images using the threshold function (lower threshold level: 7; upper threshold level: 255).
5. The “Analyze Particles” function was applied to the processed images for droplet detection (size: 0.0005–Infinity; circularity: 0.70–1.00). The droplet information for each frame was then displayed.

To realize the difference between the processed and unprocessed images in a representative offline experiment, please refer to Figure S7.

### 2.6. Online Setup

All online analyses were done on a triple quadrupole mass spectrometer (LCMS-8030; Shimadzu). The ESI source was operated in the positive-ion mode. The sample solution was injected into the ESI capillary (same as in the offline setup) using a syringe pump (serial no. D103945; KD Scientific, Holliston, MA, USA) with a flow rate of 30 μL min^−1^. Most of the analyses were carried out with a fixed voltage, except for the voltage optimization test. In that test, a 4-min stepwise voltage ramp was applied to the ESI capillary. Voltage was not applied to the ESI capillary in the first minute. In the second minute, it was +4.0 kV; in the third minute, it was +4.5 kV; and in the fourth minute, it was +5.0 kV. MS inlet voltage was maintained at 0 V throughout the experiment. The distance between the ESI capillary axis and the MS inlet was fixed at 10 mm, whereas the horizontal offset was maintained at 1.5 mm unless noted otherwise. The flow rate of nebulizing gas (nitrogen) was 2.0 L min^−1^, while the flow rate of drying gas (nitrogen) was 15.0 L min^−1^ (unless noted otherwise). The temperatures of the desolvation line and heat block were set to 250 °C and 400 °C, respectively. In the online experiment, in addition to the dielectric plate mentioned above, we also used two different designs of dielectric plates (99.6% aluminum oxide; Xide Technology, Taoyuan, Taiwan; Figure S2B,C). The photograph of each design is shown in Figure S3A. In each case, a standard analysis (without the dielectric plate) was conducted first (Figure S8A). Subsequently, the original sampling cone was removed from the front of the MS inlet and replaced with the dielectric plate. The dielectric plate was fixed with two ceramic screws (Figure S8B). Multiple reaction monitoring (MRM) mode with specific transitions was used to observe the signal changes (Figure 1B).

### 2.7. Online Experiment Data Analysis

Raw data of the extracted ion currents were exported from the LabSolutions software (version 5.97; Shimadzu) to ASCII files. Subsequently, these files were imported to Excel software (version 2019; Microsoft, Redmond, WA, USA). Three replicate datasets were averaged, and standard deviations were represented as error bars. The results for both datasets with and without dielectric plates were plotted using OriginPro software (version 8.5; OriginLab, Northampton, MA, USA). To compare the signal gain by dielectric plate numerically, the enhancement factors (*EF*s) were calculated for the online experiments:

(4) $$EF=\frac{I}{I_{0}}$$

where *I*_0_ is the averaged signal intensity for one minute without a dielectric plate (unless noted otherwise), while *I* is the averaged signal intensity for one minute with a dielectric plate (unless noted otherwise).

## 3. Results and Discussion

### 3.1. Proof-of-Concept

An offline experiment was conducted to verify the existence of charge buildup on the dielectric material surface following exposure to the electrospray plume (Figure S6A). As the time of exposure to the electrospray plume increased, the amount of detected charge increased, and the surface charge density value became saturated at ∼8 × 10^−5^ C m^−2^ after 3 min exposure (Figure S6B). Meanwhile, for the trials without electrospray, the surface charge density value was below zero. This might be caused by dielectric polarization within the dielectric plate in the presence of the energized electrospray capillary. When a positive electric field is applied near the dielectric plate, the dipoles within the aluminum oxide are rearranged, causing charge displacement. As a result, the material surface—which was placed in an external electric field—showed a temporary negative charging. No peak was recorded when the high voltage was turned off. This result shows that the dielectric material accumulates charges during exposure of its surface to the electrospray plume.

**Figure 2:**
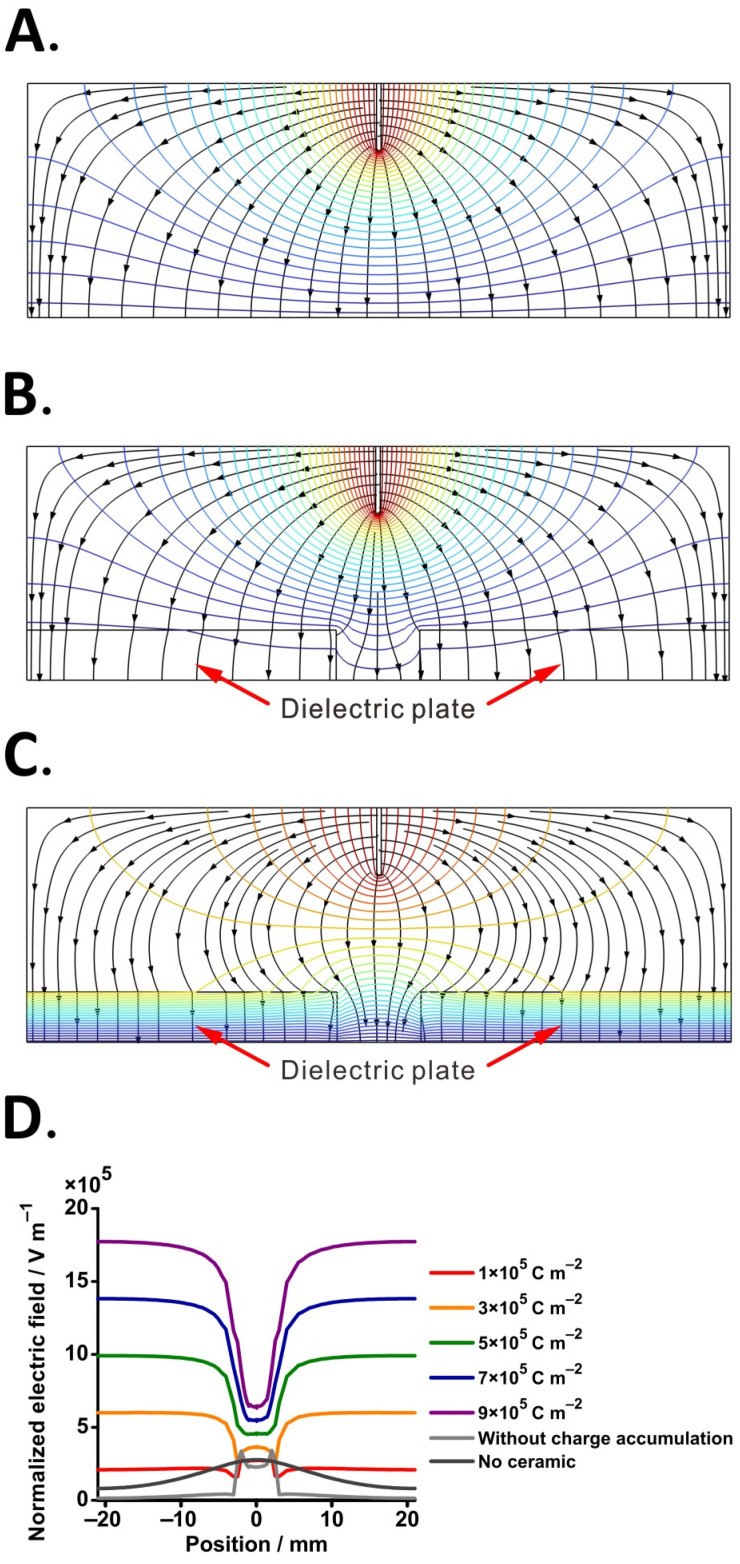
Numerical simulations of the electric field: (**A**) without dielectric plate; (**B**) with dielectric plate—without charge accumulation; (**C**) with dielectric plate—with charge accumulation (surface charge density = 5 × 10^−5^ C m^−2^); (**D**) electric field distribution plot of different surface charge densities on the dielectric plate surface. Color contour lines: equipotential line. Black lines with arrows: electric field streamlines.

Next, in order to verify the influence of dielectric material on the electric field distribution in front of the MS inlet, we performed simulations of the electric field and electric potential considering three scenarios: (A) control—standard ESI without dielectric plate; (B) ESI with dielectric plate—before charge accumulation; (C) ESI with dielectric plate—after charge accumulation (Figure 2). It can be seen that charge buildup on the dielectric surface has an effect on the curvature of the electric field, leading to the presence of a dense electric field on the exposed grounded zone (Figure 2C). Moreover, the potential distribution changes in the presence of the dielectric plate with accumulated charges. Equipotential lines near the orifice are initially concave (Figure 2B), whereas when the charge accumulates, equipotential lines are convex with respect to the ESI capillary (Figure 2C). These simulation results are in agreement with the results reported by Wei et al. [65]. It is known that ions or even charged droplets move in the orthogonal direction to the equipotential line in the gas flow-free region [31,57,66]. Therefore, the simulation of electric field distribution provides initial evidence to support the proposed idea.

To explore the influence of charge accumulation level on the electric field distribution, three different scenarios were simulated: (i) standard ESI without dielectric plate, (ii) ESI with dielectric plate without charge accumulation, and (iii) ESI with dielectric plate with five different surface charge density values. A cut line was defined on the dielectric surface, and the electric field intensities along the cut line were plotted for different conditions (Figure 2D). Clearly, the presence of the dielectric plate affects the electric field in front of the inlet regardless of charge density in the studied range (1 × 10^−5^–9 × 10^−5^ C m^−2^; Figure 2D). As shown in Figure 2D, the electric field distribution trend in the three scenarios shows a big difference. When no dielectric plate was present, the electric field value gradually increased from edge to middle (Figure 2D, black color line), while the addition of a dielectric plate without charge accumulation decreased the electric field near the covered area (Figure 2D, gray color line). For charge accumulation with a lower surface charge density (Figure 2D, red color line), it can be observed that the trend is more similar to that of the dielectric plate without charge accumulation. When the surface charge density increases to 3 × 10^−5^ C m^−2^, the electric field at the orifice is lower than the area covered by the dielectric plate. As the amount of accumulated charge increases, the gap of the electric field between the dielectric material surface and the orifice in the same cut line increases.

### 3.2. Offline Experiment

To investigate the possibility of focusing an electrospray plume with a dielectric material further, we performed a series of offline tests. In these tests, electrospray was directed toward the conductive surface of the ITO glass, which acted as a counter electrode (Figure 1A). In some of these tests, the conductive surface was additionally covered with ceramic plates with orifices of different sizes. The electrospray solution contained a fluorescent compound (fluorescein). The charged droplets impinging onto the ITO surface were observed by the optical system positioned on the opposite side of the ITO glass. The raw images were processed; 30 frames were analyzed from each replicate, and six replicates were combined (180 frames in total). This way, the positions of the electrosprayed droplets could be recorded.

**Figure 3:**
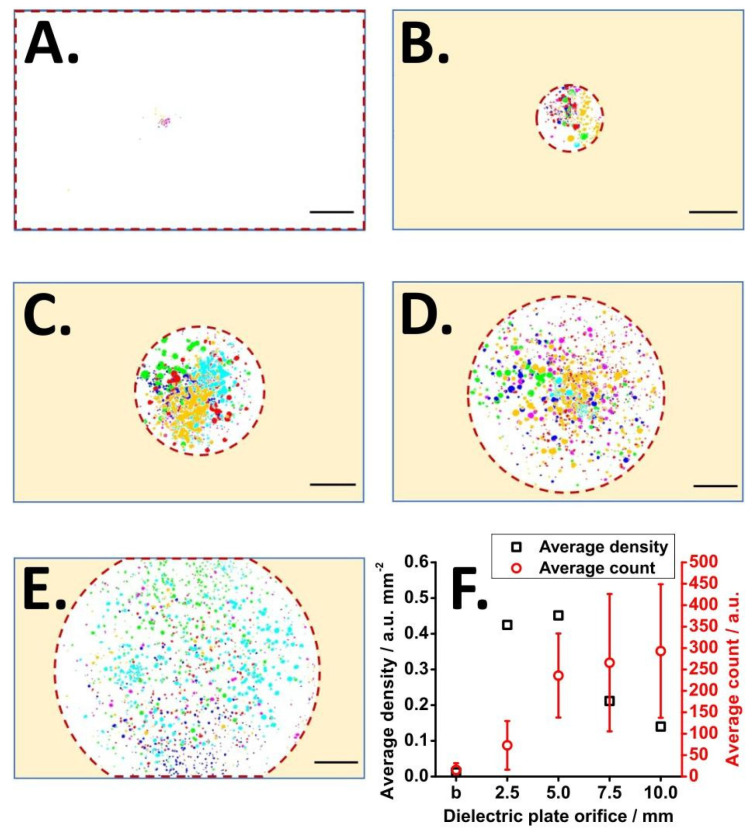
Focusing effect on different diameters of dielectric plate orifice: (**A**) no dielectric plate; (**B**) orifice diameter = 2.5 mm; (**C**) orifice diameter = 5.0 mm; (**D**) orifice diameter = 7.5 mm; (**E**) orifice diameter = 10.0 mm; (**F**) plot of droplet density and droplets count as a function of orifice size. The result corresponding to the variant without a dielectric plate is labeled as ‘b’ on the horizontal axis. The distance between the ESI capillary tip and the ITO surface is 10 mm. ESI voltage: +4.5 kV. Sample: 7.5 × 10^−5^ M fluorescein in 75% (*v*/*v*) methanol in water + 0.2% (*v*/*v*) ammonium hydroxide. The sample was injected by a hydrodynamic pump with a flow rate of ~10 µL min^−1^. The circles with different colors in (**A**–**E**) correspond to the replicate analyses carried out on different days. The red dashed line indicates the boundary of the area that was not covered by the dielectric plate. Scale bar: 2 mm.

Figure 3A–E and Movie S1 show the distributions of the electrosprayed droplets on the ITO glass surface when dielectric plates with different orifices were used.

It is evident that the droplet trajectories were affected significantly by the presence of the dielectric. To further analyze the imaging results, the density (*d*_droplet_) of the deposited droplets was calculated based on the following equation:

(5) $$d_{droplet}=\frac{n}{A_{r}}$$

where *n* is the number of droplets detected within the area (*A*_r_) highlighted with a red dashed line in each image. Interestingly, the spot density is the highest for the 5 mm orifice, which proves the focusing effect of the dielectric plate (Figure 3F). Although the greatest number of droplets was observed using the dielectric plate with 10 mm orifice, the density of the droplets was relatively low, which means that the droplets were dispersed in a larger area. The non-monotonous characteristics of the density-vs-orifice size indicate that the observed effect is not merely due to the blocking of the droplets relayed onto the ITO glass surface. The difference in plume size after placing a 5 mm orifice dielectric plate also reveals that the dielectric plate led to a focusing effect instead of a blocking effect. Based on this result, it seems that the plate with 5 mm orifice can provide the maximum focusing. A side view of the plume also illustrates that the plume is collimated in the presence of the dielectric plate with an orifice (Figure S9).

### 3.3. Online Experiment

The influence of the dielectric plate positioned in front of the MS orifice on the signal intensity was verified. The key parameters that influence MS signal intensity—such as ESI voltage (Figure S10), sampling flow rate (Figure S11), drying and nebulizing gas flow rate (Figure S12), as well as solvent composition (Figure S13)—were optimized. It was found that ESI voltage and sample flow rate do not have a big effect on *EF* (Figures S10 and S11). In contrast, the flow rate of both drying and nebulizing gas influenced signal enhancement (Figure S12). That is because the flow rates (velocities) of these gasses can affect the desolvation process and droplet velocities [6,67]. These results suggest that 15.0 L min^−1^ and 2.0 L min^−1^ are the optimum flow rates for drying gas and nebulizing gas, respectively. Furthermore, the effect of solvent composition on signal enhancement was also observed. We found that in both methanol and ethanol solvents, higher *EF* was observed in the presence of the organic solvent (Figure S13A,B), while the addition of formic acid can increase signal intensities in both cases (with dielectric plate and without dielectric plate; Figure S13C,D).

**Figure 4:**
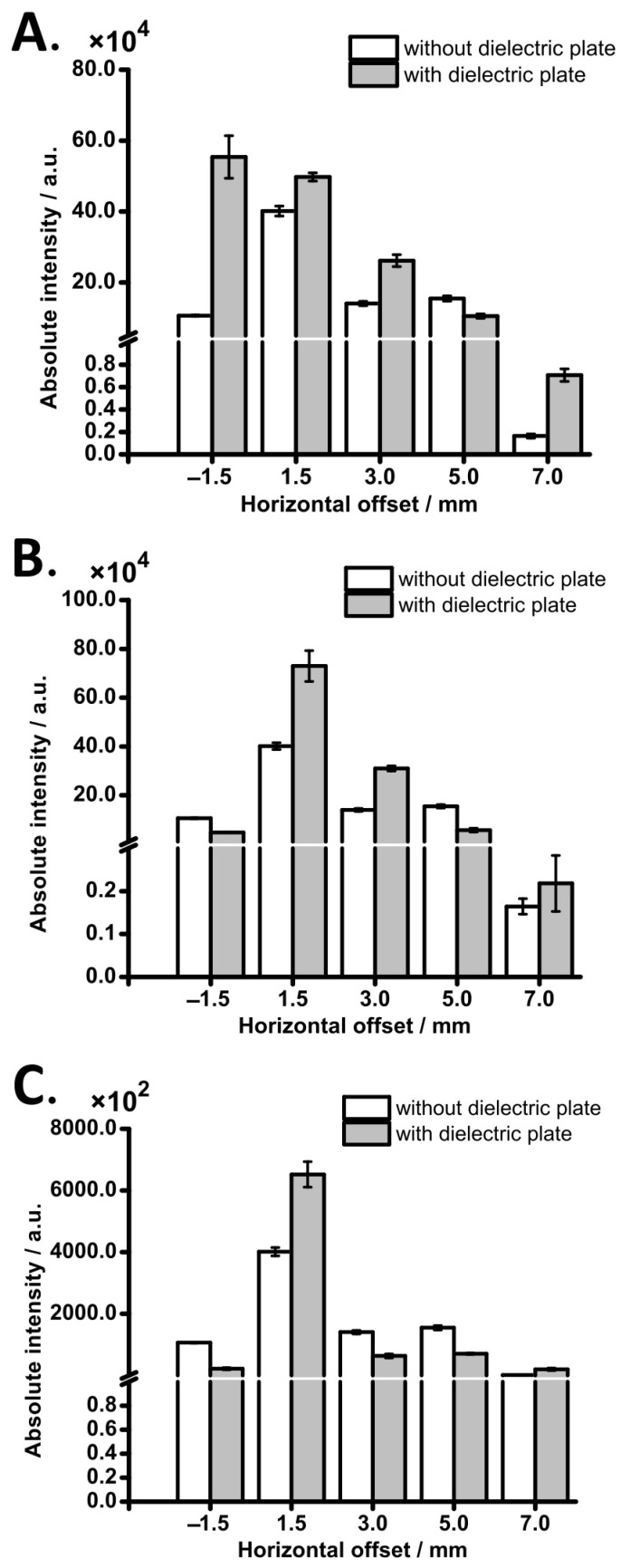
Signal enhancement effect in different designs of the dielectric plate. (**A**) Dielectric plate without cone-shaped inlet; (**B**) dielectric plate with cone-shaped inlet, without drying gas holes; (**C**) dielectric plate with cone-shaped inlet, with drying gas holes. ESI voltage: +4.5 kV. Sample: 15 μM acetaminophen in 50% (*v*/*v*) aqueous ethanol solution. Flow rate: 30 μL min^–1^. Three replicate measurements were performed.

In the next stage of the study, the effect of different orifice sizes on MS signal intensity was investigated. We found that 5 mm orifice provides the largest enhancement and lowest relative standard deviation (RSD) of MS signal intensity (Table S1), which is consistent with the offline experiment described above (Figure 3F). We further compared the performances of the three types of dielectric plate: (A) flat dielectric plate without conical feature—same as the one used in the offline experiment and the optimization experiments described above (orifice, 5 mm; Figure S2A); (B) dielectric plate with conical feature (orifice, 5 mm; Figure S2B); (C) dielectric plate with conical feature and six holes for drying gas (central hole, 2 mm; drying gas holes, 1 mm; Figure S2C). Among these plates, Plate C has a geometry that is very close to the original stainless steel sampling cone of the mass spectrometer. The alteration of signal intensity for different plates and distances of electrospray capillary from the MS inlet is depicted in Figure 4. It can be seen that in 9 out of 15 cases, there was a signal enhancement after incorporating the dielectric plate, which proves that dielectric plates—placed in front of the MS inlet—can enhance MS signals.

Interestingly, the signal gain was affected not only by the plate geometry but also by the horizontal offset between the ESI capillary and the MS inlet. In the case of negative offsets (*d* = −1.5 mm), the presence of the cone feature in the dielectric plate has a negative effect on the signal. Only plate A—which is devoid of the conical feature—shows signal enhancement in this case. In the case of the default offset (*d* = 1.5 mm), enhancements were observed for all the tested dielectric plates, although the values were relatively low. It is possible that in this position, the airflow near the MS orifice mainly contributes to the MS signal, and the dielectric plate only assists the ions to concentrate near the MS inlet. However, the highest absolute intensity in plate B leads to the conclusion that increasing the exposed area of the ground electrode yields higher MS signals. This conclusion was also verified in the case of the 3 mm offset, where signal enhancement was observed only for plates A and B. Signals decreased drastically when the offset values were 5 mm or above. In the case of the largest offset (*d* = 7 mm), although the absolute signal intensity was very low, the dielectric plate contributed to an increase in MS signal intensity. Thus, the dielectric plate shows an effect in the case of larger distances between the ESI emitter and the MS inlet, indicating an improvement in droplet or ion transmission efficiency.

Repeatability and reproducibility results are presented in Table S2 and Figure S14. Most of the conditions provide acceptable repeatability of *EF*, showing RSDs ranging from 2.8 to 18.2%. In the case of reproducibility, RSDs range from 18.9 to 153.7%. From the reproducibility results, we observed that high RSDs were obtained when the distance between the ESI capillary and ground electrode was −1.5 mm. Increasing the electrostatic force effect by maintaining a short distance (i.e., negative offset) between the ESI capillary and ground electrode does not provide significant advantages for signal enhancement in this method. To solve this issue, isotopically labeled standards can be used to minimize the effects of inter-day signal variability [68,69]. Relationships between ion intensities and analyte concentrations were plotted in Figure S15. The enhancement effect was distinct in plates B and C with the aid of longer distance, showing that the plate geometry and distance are the two main attributes affecting *EF* in this study.

Subsequently, this method was tested in analyses of various compounds on the triple quadrupole mass spectrometer. Compounds were selected to cover a wide range of analyte properties, such as *m*/*z*, logP, p*K*_a_, and polar surface area, as these analyte properties can influence ESI efficiency [15,70]. The test was carried out with all three dielectric plates (Table 1) and used two horizontal offsets, 1.5 mm and 7 mm. These two offsets provided the highest signal intensity and the highest *EF*, respectively (*cf.* Figure 4, Table S2). The obtained results indicate that most of the analytes show a moderate signal enhancement with all dielectric plates in the default horizontal distance (*d* = 1.5 mm). Meanwhile, for a longer distance (*d* = 7 mm), it could be clearly seen that only plate C provides an enhancement for all analytes, especially for cloxacillin, with an *EF* of ~326. This shows that the conical feature with drying gas holes made of dielectric material shows an obvious effect at larger distances. Since both signal intensity and *EF* were significantly affected by the distance between the ESI capillary and MS inlet, this method may become an alternative choice for some studies to compensate for the signal loss caused by increasing the distance. Moreover, it was demonstrated that a high percentage of droplets is aspirated into MS vacuum stages, and this might lead to other unwanted consequences, such as chemical noise, contamination, and other complex interactions [71]. Thus, to extend the usage period of the instrument, it is crucial to reduce the number of droplets flowing into MS. While increasing the distance extends the desolvation time, this idea may be considered as a new way to maintain instrument lifetime without sacrificing signal intensity. Apart from this, it is worth noting that the ions of the same compound with different charge numbers also provide different signal enhancements. According to Table 1, higher *EF* was observed for singly charged ions of the compounds that also exhibit multiply charged ions (angiotensin II, HPF2, HPF3). Since space charge repulsion was found to be one of the ion loss mechanisms in previous studies [42,72], it is possible that the space charge effect of highly charged ions leads to ion loss, causing the observed trend. These results may also explain why the solvent with formic acid did not contribute to a large signal enhancement, as formic acid facilitates charging (Figure S13). Furthermore, to investigate the enhancement trend of varied charge states, two proteins (ubiquitin and cytochrome *c*) were tested. For both proteins, signal intensities of each charge state were acquired using a single ion monitoring mode. Six conditions (three dielectric plates, two distances) were tested, and the *EF*s were displayed using a heatmap (Figure S16). In both proteins, the highest enhancement of all charge states was observed in plate C, regardless of the distance. The *EF* is proportional to the charge state in a 1.5 mm offset (Figure S16A,C). Moreover, in the case of plate C, increasing the offset affects the signal enhancement of each charge state. The proportionality between the charge state and *EF* observed in 1.5 mm was not observed in 7 mm (Figure S16B,D). The highest *EF* was not observed from the highest charge state but rather from a lower charge state (+9 for ubiquitin and +10 for cytochrome *c*).

### 3.4. Final Considerations

The feasibility of using a dielectric material to increase MS performance was verified in both offline and online experiments. However, only moderate signal enhancement was observed in the online experiments. The *EF* was lower than expected based on the offline experiment. The offline experiments showed focusing of large microdroplets, whereas the online experiments determined the enhancement of MS signals. To the best of our knowledge, the signal intensities of ions in online experiments are primarily associated with nanodroplets rather than microdroplets. This may result in a relatively lower level of focusing when compared to the observations from the offline experiments. Moreover, some negative effects of nebulizing gas and drying gas flow on ion transmission in the ambient ion source have been previously reported [73,74]. Therefore, the hydrodynamic effect should also be considered as one of the factors that may contribute to a lower *EF*. When using this method, we did not increase the frequency of instrument maintenance. Nevertheless, there is a possibility that the prolonged use of this method may lead to contamination due to the accumulation of sample matrix in the mass spectrometer’s inlet. Note that the ceramic plates were cleaned before each experiment (placed in pure water and sonicated for 15 min).

## 4. Conclusions

We have disclosed a simple idea of enhancing the MS signals by placing a dielectric plate with an orifice in front of the MS inlet. The dielectric plate changes the distribution of the electric field, which leads to the focusing of charged particles produced by the electrospray. The idea was initially verified in an offline experiment in which electrospray droplets impinged on the conductive glass. The obtained images revealed significant focusing of the charged droplets. The idea was further verified in an online experiment using a triple quadrupole mass spectrometer. Placing the dielectric plate in front of the MS orifice led to a moderate signal enhancement in the case of some analyzed compounds, while a decrease in signal was seen in the case of other compounds. The signal enhancement was particularly high when the orthogonal electrospray emitter was moved away from the MS inlet. However, when using the default distance, the signal enhancements were relatively low; thus, there is room for improvement in the follow-up work. While we employed only one type of mass spectrometer here, the idea of electrospray droplet focusing with the dielectric plate can be further explored on other mass spectrometers with the atmospheric pressure interface.

## Figures and Tables

**Table 1:** *EF*s for various tested analytes (*n* = 3). Concentration: 15 μM. Solvent: 50% (*v*/*v*) aqueous ethanol solution.

Compound	MRM Transition	Horizontal Offset = 1.5 mm			Horizontal Offset = 7 mm		
		Plate A	Plate B	Plate C	Plate A	Plate B	Plate C
Acetaminophen	152→110	1.24 ± 0.06	1.82 ± 0.19	1.62 ± 0.08	4.35 ± 0.77	1.31 ± 0.29	12.18 ± 1.48
Alanine	90→44	1.17 ± 0.03	1.14 ± 0.06	1.27 ± 0.06	0.04 ± 0.00	1.86 ± 0.60	4.40 ± 0.46
Angiotensin II (singly charged)	1047→110	1.86 ± 0.12	1.36 ± 0.13	1.47 ± 0.18	0.05 ± 0.01	10.52 ± 0.90	30.70 ± 2.98
Angiotensin II (doubly charged)	524→70	1.83 ± 0.16	1.39 ± 0.11	1.65 ± 0.20	0.09 ± 0.02	3.80 ± 0.40	10.19 ± 0.72
Citrulline	176→159	1.33 ± 0.09	1.35 ± 0.06	1.26 ± 0.04	1.08 ± 0.17	4.85 ± 0.56	10.10 ± 1.97
Cloxacillin	458→182	1.21 ± 0.02	1.39 ± 0.09	1.50 ± 0.09	0.54 ± 0.08	103.91 ± 23.75	325.69 ± 42.40
Glutathione	308→179	1.47 ± 0.06	1.16 ± 0.01	1.29 ± 0.03	0.27 ± 0.05	6.99 ± 0.55	13.36 ± 0.69
Glycine	76→30	0.81 ± 0.06	1.14 ± 0.06	1.32 ± 0.05	0.15 ± 0.02	2.26 ± 0.61	4.16 ± 0.33
Glycine-Histidine peptide	213→156	1.52 ± 0.06	1.40 ± 0.06	1.28 ± 0.05	0.61 ± 0.14	6.88 ± 0.79	24.94 ± 3.70
HPF1	400→263	1.34 ± 0.05	1.42 ± 0.08	1.48 ± 0.09	0.17 ± 0.04	10.03 ± 0.56	21.62 ± 0.37
HPF2 (singly charged)	781→364	1.12 ± 0.01	1.27 ± 0.01	1.02 ± 0.01	2.19 ± 0.53	113.68 ± 21.56	294.69 ± 96.95
HPF2 (doubly charged)	391→110	1.16 ± 0.02	1.32 ± 0.03	0.90 ± 0.04	0.05 ± 0.00	7.43 ± 0.47	12.30 ± 1.91
HPF3 * (singly charged)	1163→110	0.86 ± 0.03	0.96 ± 0.03	1.02 ± 0.05	0.00 ± 0.00 **	201.49 ± 196.22	622.28 ± 677.48
HPF3 (doubly charged)	582→110	1.00 ± 0.05	0.94 ± 0.05	0.94 ± 0.01	0.15 ± 0.01	4.23 ± 0.47	7.50 ± 0.11
HPF3 (triply charged)	388→110	1.11 ± 0.14	1.01 ± 0.14	1.17 ± 0.12	0.28 ± 0.03	5.64 ± 0.83	8.29 ± 0.85
Lysine	147→84	1.65 ± 0.18	1.75 ± 0.02	1.48 ± 0.07	0.18 ± 0.04	2.24 ± 0.40	6.11 ± 0.24
Tryptophan	205→146	1.12 ± 0.06	1.15 ± 0.08	1.09 ± 0.06	0.76 ± 0.02	0.05 ± 0.05	15.42 ± 1.00

* In 7 mm, the signal intensity of one of the replicates without a dielectric plate was equal to zero, leading to calculation error. As a result, the *EF* of HPF3 (singly charged) in 7 mm was obtained by two replicates, indicating the average and spread of the data. ** A final averaged *EF* of zero was obtained due to the zero absolute intensity observed in two of the replicates in plate A. One replicate was excluded due to zero value in the control experiment (*I*_0_, without dielectric plate). *EF* was calculated by dividing the averaged signal for 1 min with a dielectric plate (*I*) over the averaged signal for 1 min without a dielectric plate (*I*_0_).

## Data Availability

The data that support the findings of this study are available from the corresponding author upon reasonable request.

## Supplementary Materials

The following supporting information can be downloaded at: https://www.mdpi.com/article/10.3390/molecules29020316/s1, Movie S1: Focusing effect on different diameters of dielectric plate orifice; Table S1: Enhancement factors (*EF*s) for different orifice sizes of dielectric plate (*n* = 3); Table S2: Analytical results for the dependency of signal intensity without and with dielectric plate on distance of electrospray emitter axis from the MS inlet; Figure S1: Schematic of tubing connections in the hydrodynamic pump (not to scale); Figure S2: Technical drawings of the three dielectric plates used in this study; Figure S3: Photographs of dielectric plates used in this study; Figure S4: Technical drawings of the customized 3D-printed ITO glass holder used in this study; Figure S5: 2D model of the off-line setup used in the simulation; Figure S6: Measurement of surface charge density on the dielectric plate after different times of electrospray durations; Figure S7: Representation of the off-line experiment result; Figure S8: Photographs of MS interface with/without dielectric plate in front view (left) and side (right) view; Figure S9: Effect of the dielectric plate on the plume shape; Figure S10: Effect of the ESI voltage on signal intensities and enhancement in the on-line experiment; Figure S11: Effect of sample flow rate to signal intensities and enhancement; Figure S12: Effect of drying gas flow rate and nebulizing gas flow rate on ion intensities in the presence of dielectric plate; Figure S13: Effect of solvent on ion intensities in the presence of dielectric plate; Figure S14: Dependency of signal intensity without and with dielectric plate on distance of electrospray emitter axis from the MS inlet; Figure S15: Influence of the analyte concentration on ion intensity at different distances with or without the dielectric plate; Figure S16: Influence of dielectric plates on signals of different proteins; Computer code.
