# Supplementary material for: Moderate Signal Enhancement in Electrospray Ionization Mass Spectrometry by Focusing Electrospray Plume with a Dielectric Layer around the Mass Spectrometer’s Orifice
Source: Molecules. 2024 Jan 8;29(2):316. doi: 10.3390/molecules29020316 (PMC10821223; doi:10.3390/molecules29020316)
Supplement: Supplementary file 1 [file molecules-29-00316-s001.zip › SI_ESI_focusing_final_Molecules.pdf]

SUPPORTING INFORMATION

Moderate Signal Enhancement in Electrospray Ionization Mass Spectrometry by Focusing Electrospray Plume with a Dielectric Layer around the Mass Spectrometer's Orifice

Zi Qing Chua,[a] Gurpur Rakesh D. Prabhu,[a] Yi-Wun Wang,[a] Chamarthi Maheswar Raju,[a] Krzysztof Buchowiecki,[a] Ochir Ochirov,[a] Decibel P. Elpa,[a] Pawel L. Urban[a,b]*

[a] Department of Chemistry, National Tsing Hua University

101, Section 2, Kuang-Fu Rd., Hsinchu, 300044, Taiwan

[b] Frontier Research Center on Fundamental and Applied Sciences of Matters,

National Tsing Hua University

101, Section 2, Kuang-Fu Rd., Hsinchu, 300044, Taiwan

* Corresponding author:

P.L. Urban (urban@mx.nthu.edu.tw)

ADDITIONAL TABLES

Table S1. Enhancement factors (*EF*s) for different orifice sizes of dielectric plate (n = 3). Plate A (flat plate without conical feature) was used in this experiment. Nebulizing gas flow rate: 2 L min^{-1}. Drying gas flow rate: 15 L min^{-1}. Sample solution: 15 μM acetaminophen in 50% (v/v) aqueous ethanol solution (MRM transition: 152→110). ESI voltage: +4.5 kV. Horizontal offset between the ESI capillary and the MS inlet: 1.5 mm. Note that this experiment was performed on a different day than the experiment presented in **Table S2**. Thus, the average *EF* (1.19) is slightly lower than the corresponding value in **Table S2** (1.23).

**Orifice size / mm**	**Average *EF***	**RSD / % (*n* = 9; 3 days; each day, *n* = 3)**
2.5	0.74	22.7
5.0	**1.19**	3.4
7.5	1.10	7.8
10.0	1.04	4.8

Table S2. Analytical results for the dependency of signal intensity without and with dielectric plate on distance of electrospray emitter axis from the MS inlet. Nebulizing gas flow rate: 2 L min^{-1}. Drying gas flow rate: 15 L min^{-1}. Sample solution: 15 μM acetaminophen in 50% (v/v) aqueous ethanol solution (MRM transition: 152→110). ESI voltage: +4.5 kV. Note that this experiment was performed on a different day than the experiment presented in **Table S1**. Thus, the average *EF* (1.23) is slightly higher than the corresponding value in **Table S1** (1.19).

**Plate**	**Horizontal offset / mm**	**Repeatability (1 day; *n* = 5)**		**Reproducibility (*n* = 9; 3 days; each day, *n* = 3)**	
		**EF mean**	**RSD / %**	**EF mean**	**RSD / %**
**A (without conical feature, orifice size: 5 mm)**	-1.5	1.89	3.2%	2.99	64.2%
	1.5	**1.23**	3.8%	1.36	18.9%
	3	1.25	5.0%	1.44	25.0%
	5	1.08	7.9%	0.87	24.1%
	7	4.72	10.8%	3.16	34.4%
**B (with conical feature, orifice size: 5 mm)**	-1.5	0.03	18.2%	0.19	119.6%
	1.5	1.04	4.3%	1.26	39.2%
	3	1.56	4.3%	1.67	27.7%
	5	1.20	3.4%	0.82	47.9%
	7	25.14	13.1%	7.00	109.0%
**C (with conical feature and 6 holes for drying gas, orifice size: 5 mm)**	-1.5	0.03	5.0%	0.08	153.7%
	1.5	1.12	2.8%	1.33	26.8%
	3	1.51	3.9%	1.03	49.3%
	5	1.46	3.7%	0.98	46.5%
	7	36.29	6.0%	18.33	62.4%

ADDITIONAL FIGURES

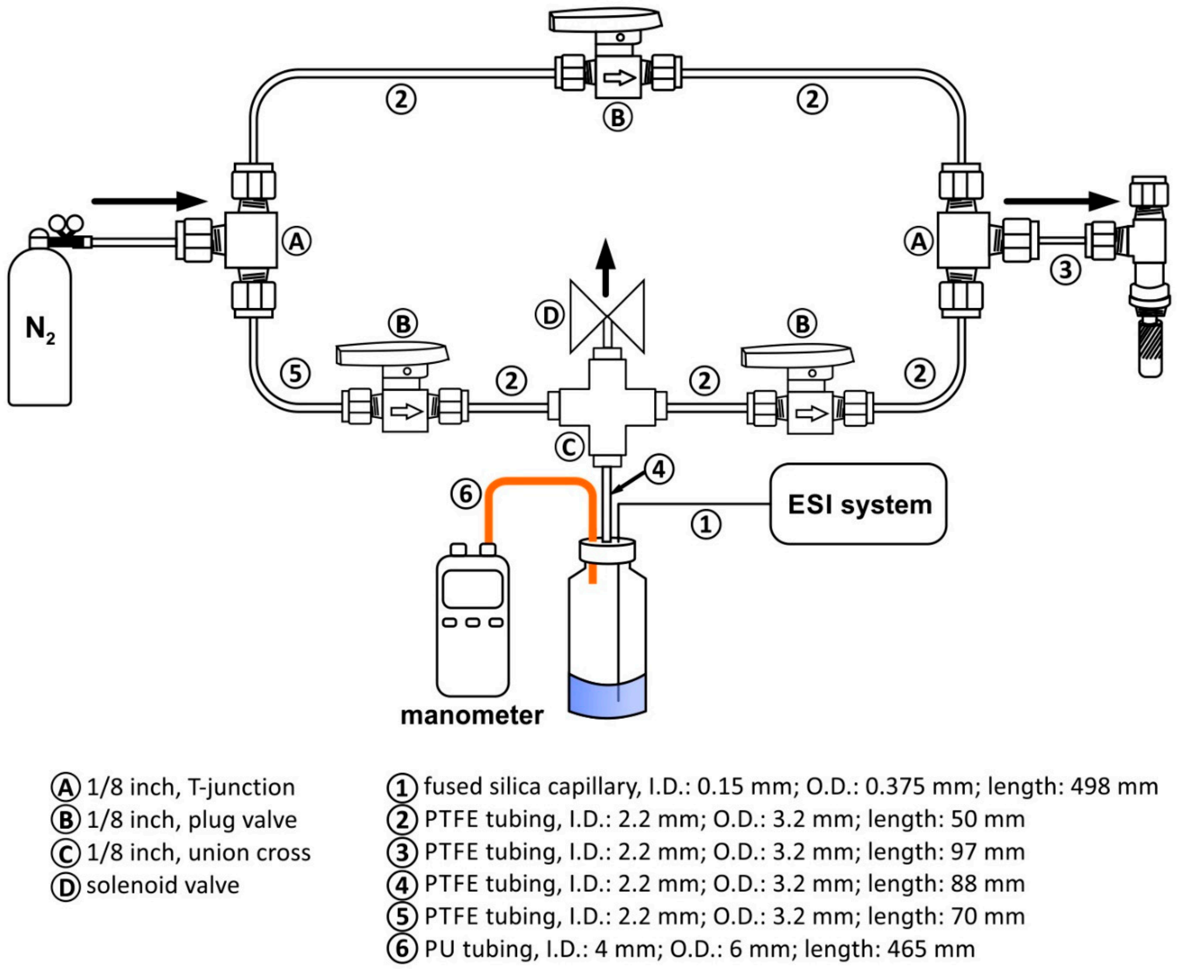

Figure S1. Schematic of tubing connections in the hydrodynamic pump (not to scale).

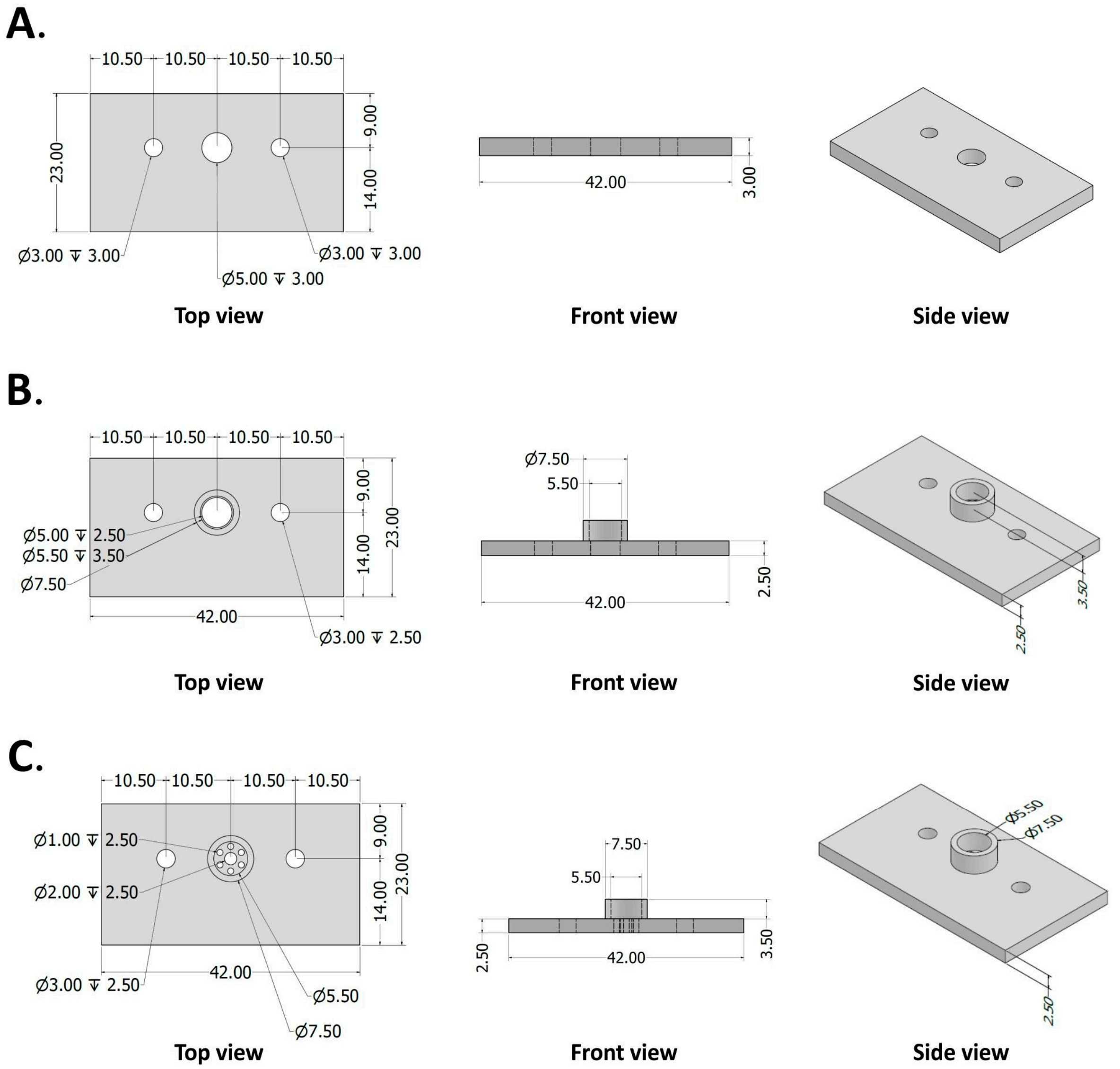

Figure S2. Technical drawings of the three dielectric plates used in this study: (A) Plate A – flat dielectric plate without conical feature; (B) Plate B – dielectric plate with conical feature; (C) Plate C – dielectric plate with conical feature and 6 holes for drying gas. Unit: mm.

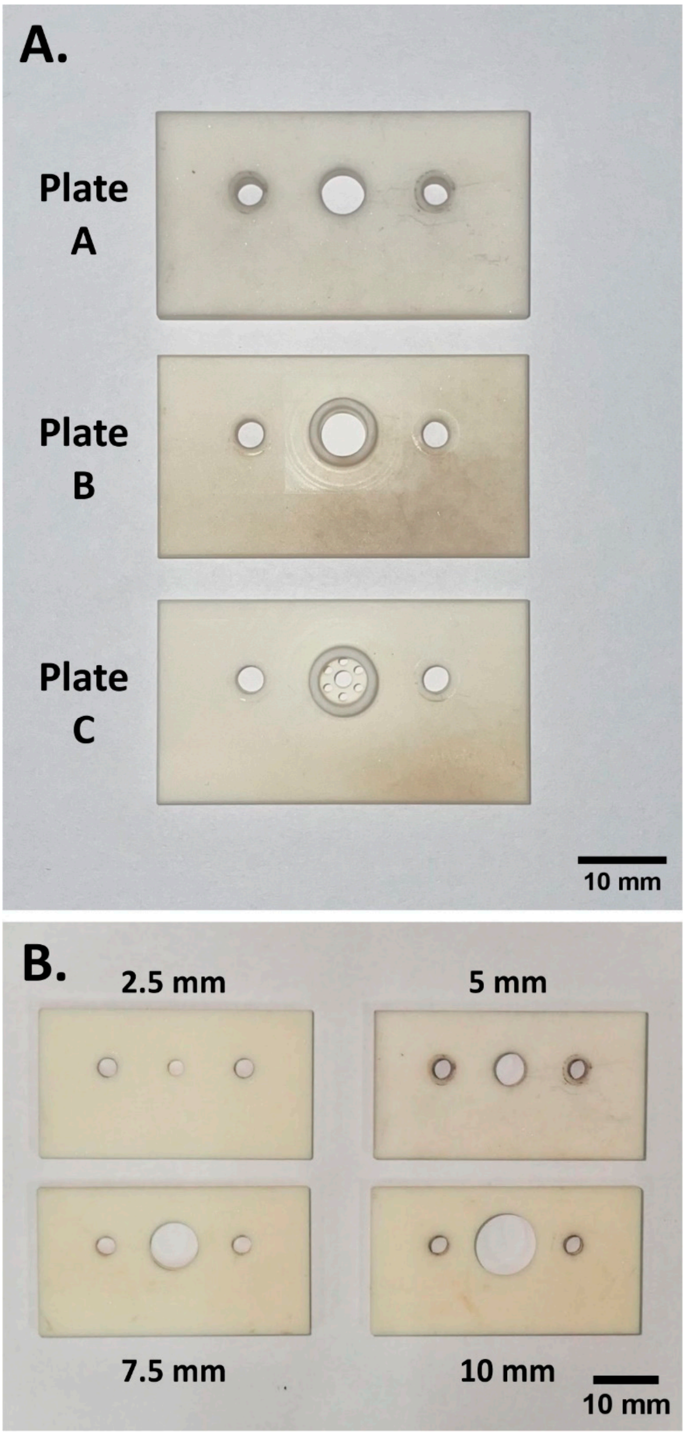

Figure S3. Photographs of dielectric plates used in this study: (A) three different designs used in this study; (B) Plate A with different orifice sizes. These plates were used to acquire data of offline experiment and **Table S1**.

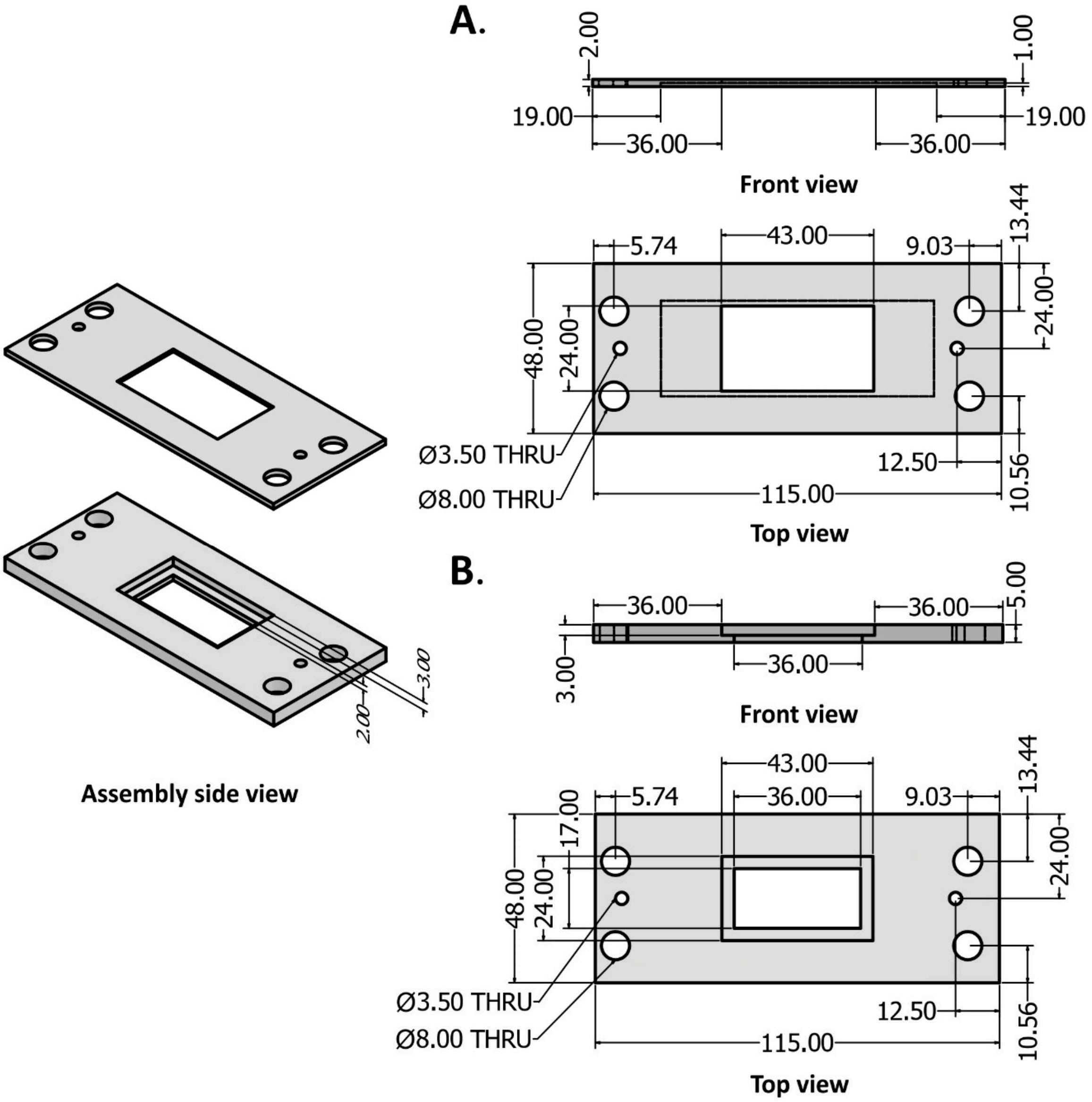

Figure S4. Technical drawings of the customized 3D-printed ITO glass holder used in this study: (A) upper layer of the holder; (B) bottom layer of the holder. For bottom layer, a groove was designed to fix the dielectric plate. Left part is the combination of two layers. Two parts of the holder were assembled by two screws.

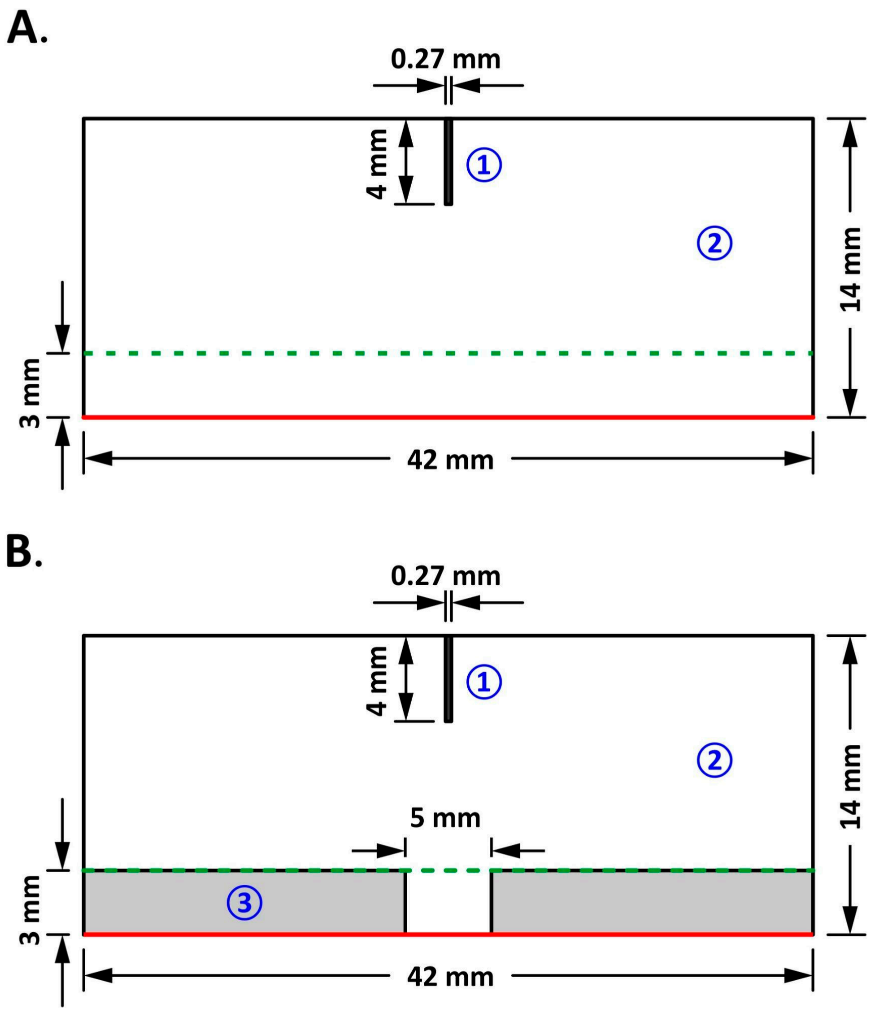

Figure S5. 2D model of the off-line setup used in the simulation: (A) without dielectric plate; (B) with dielectric plate. The red line indicates the grounded surface. Blue numbers: (1) ESI capillary; (2) ambient atmospheric air region; (3) dielectric plate. The green dashed line was defined as the cut line for electric field measurement. The origin in this simulation is defined by the center of the red line.

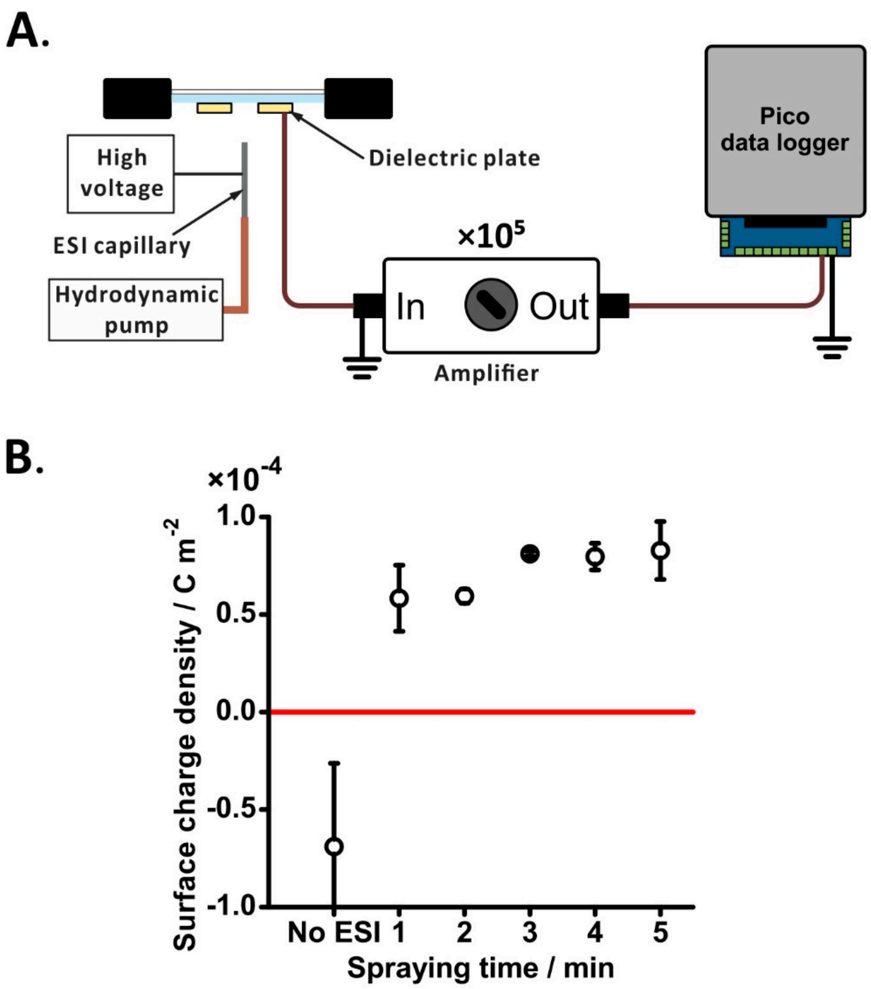

Figure S6. Measurement of surface charge density on the dielectric plate after different times of electrospray durations: (A) schematic of wiring connection for surface charge density measurement; (B) surface charge density *vs.* spraying time (n = 3). Note that for blank (no solution sprayed and high voltage applied), no peak was detected.

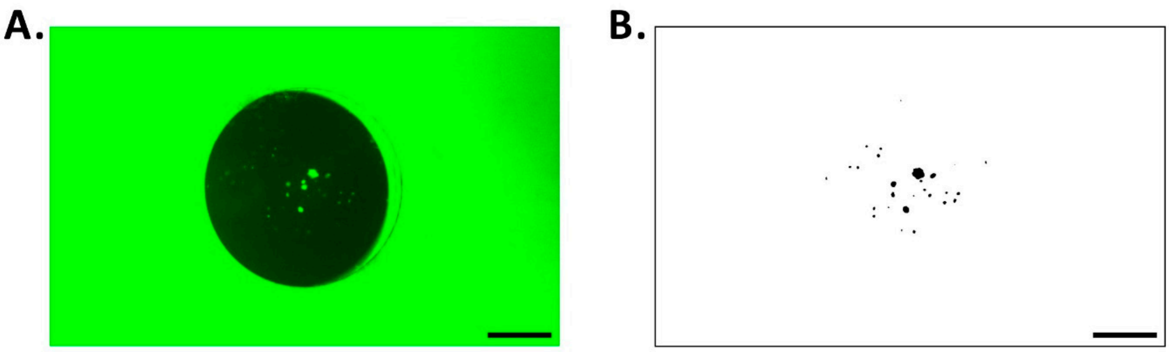

Figure S7. Representation of the off-line experiment result: (A) unprocessed image; (B) processed image. Diameter of the orifice: 5 mm. Scale bar: 2 mm.

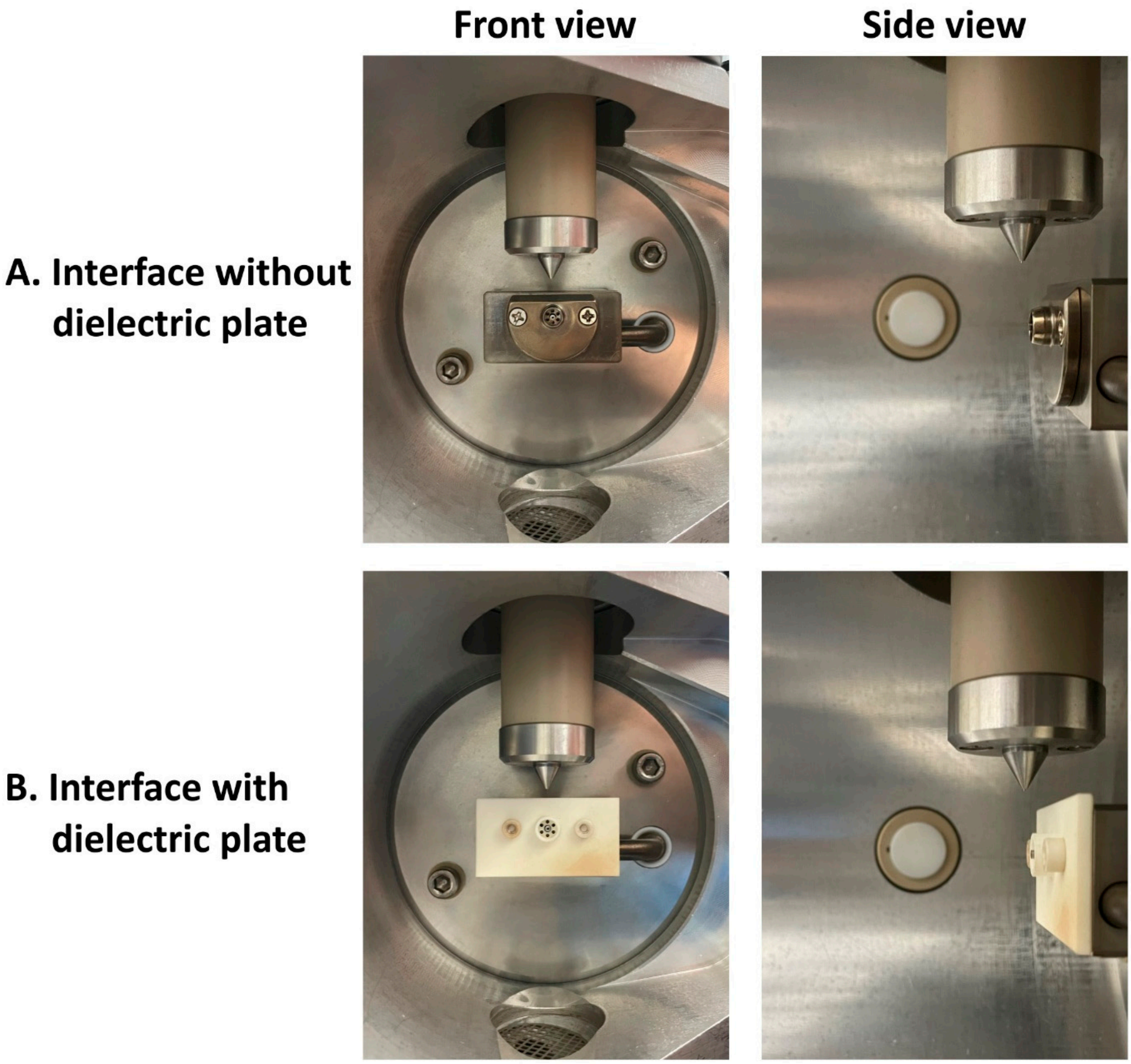

Figure S8. Photographs of MS interface with/without dielectric plate in front view (left) and side (right) view: (A) without dielectric plate; (B) with dielectric plate (Plate C). In (B), ceramic screws were used to fix the dielectric plate.

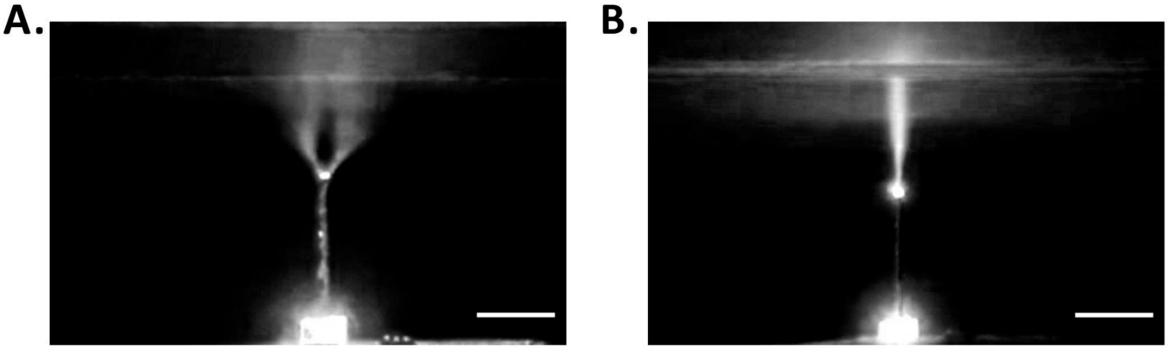

Figure S9. Effect of the dielectric plate on the plume shape: (A) without dielectric plate; (B) with dielectric plate (plate A). Distance between ESI capillary tip and ground electrode: 10 mm. Voltage: +4.5 kV. Diameter of the dielectric plate orifice: 5 mm. Scale bar: 2 mm.

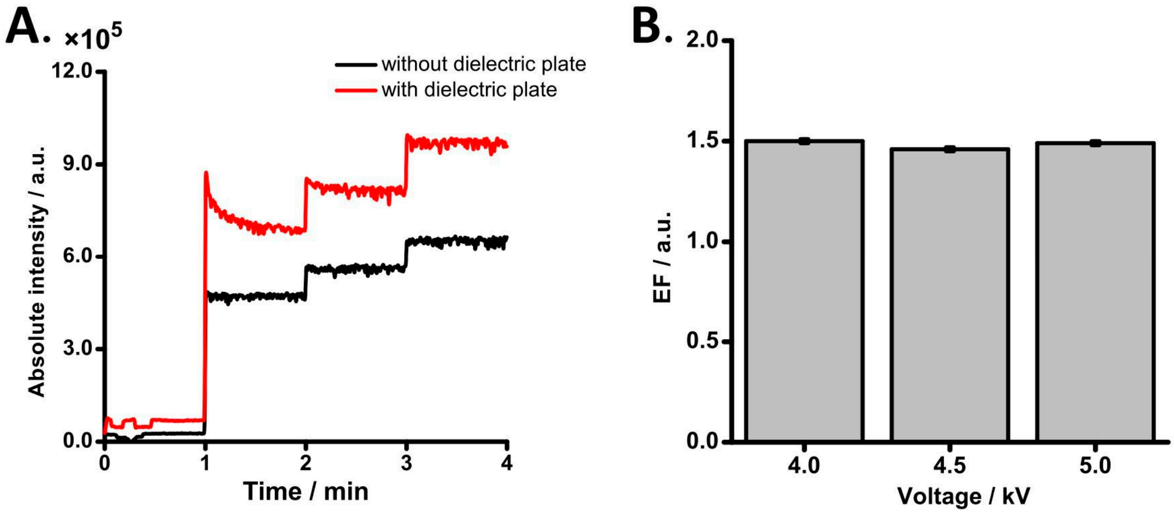

Figure S10. Effect of the ESI voltage on signal intensities and enhancement in the on-line experiment: (A) voltage scan with/without dielectric plate. In the voltage ramp experiment, data were averaged within the time interval of 0.6 min (0.2-0.8 min for 0 kV, 1.2-1.8 min for 4.0 kV, 2.2-2.8 min for 4.5 kV, 3.2-3.8 min for 5.0 kV), and standard deviation was obtained for the same time range. Line was plotted with the mean of the triplicates; (B) *EF* values of each applied voltage. Horizontal offset: +1.5 mm. Drying gas flow rate: 15 L min^{-1}. Nebulizing gas flow rate: 2 L min^{-1}. Sample solution: 15 μM acetaminophen in 50% (v/v) aqueous ethanol solution. MRM transition: 152→110. Replicates, $n = 3$.

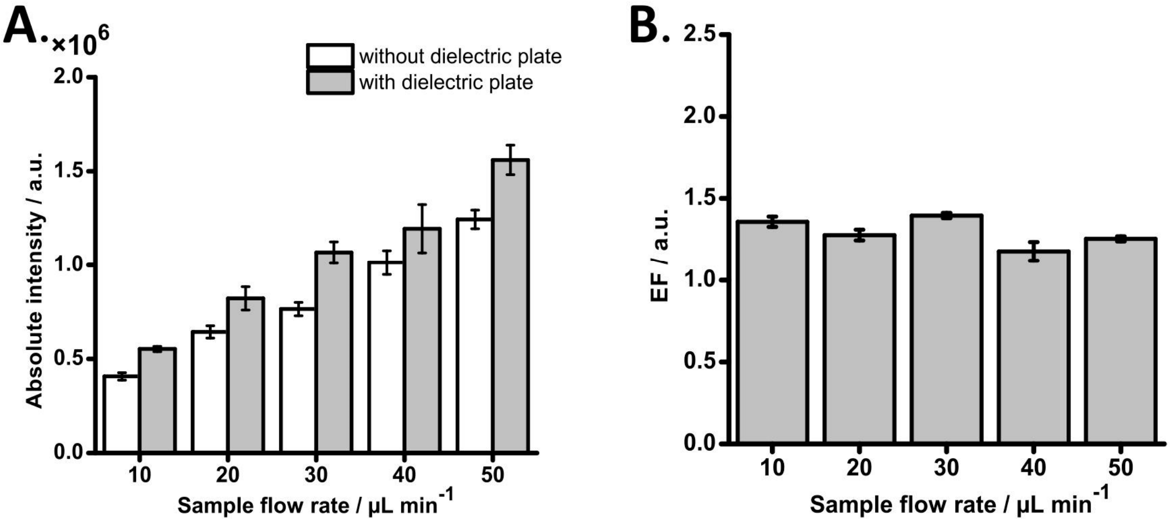

Figure S11. Effect of sample flow rate to signal intensities and enhancement. Horizontal offset between ESI capillary and MS inlet: +1.5 mm. Drying gas flow rate: 15 L min^{-1}. Nebulizing gas flow rate: 2 L min^{-1}. Sample solution: 15 µM acetaminophen in 50% (v/v) aqueous ethanol solution (MRM transition: 152→110). Replicates, $n = 3$.

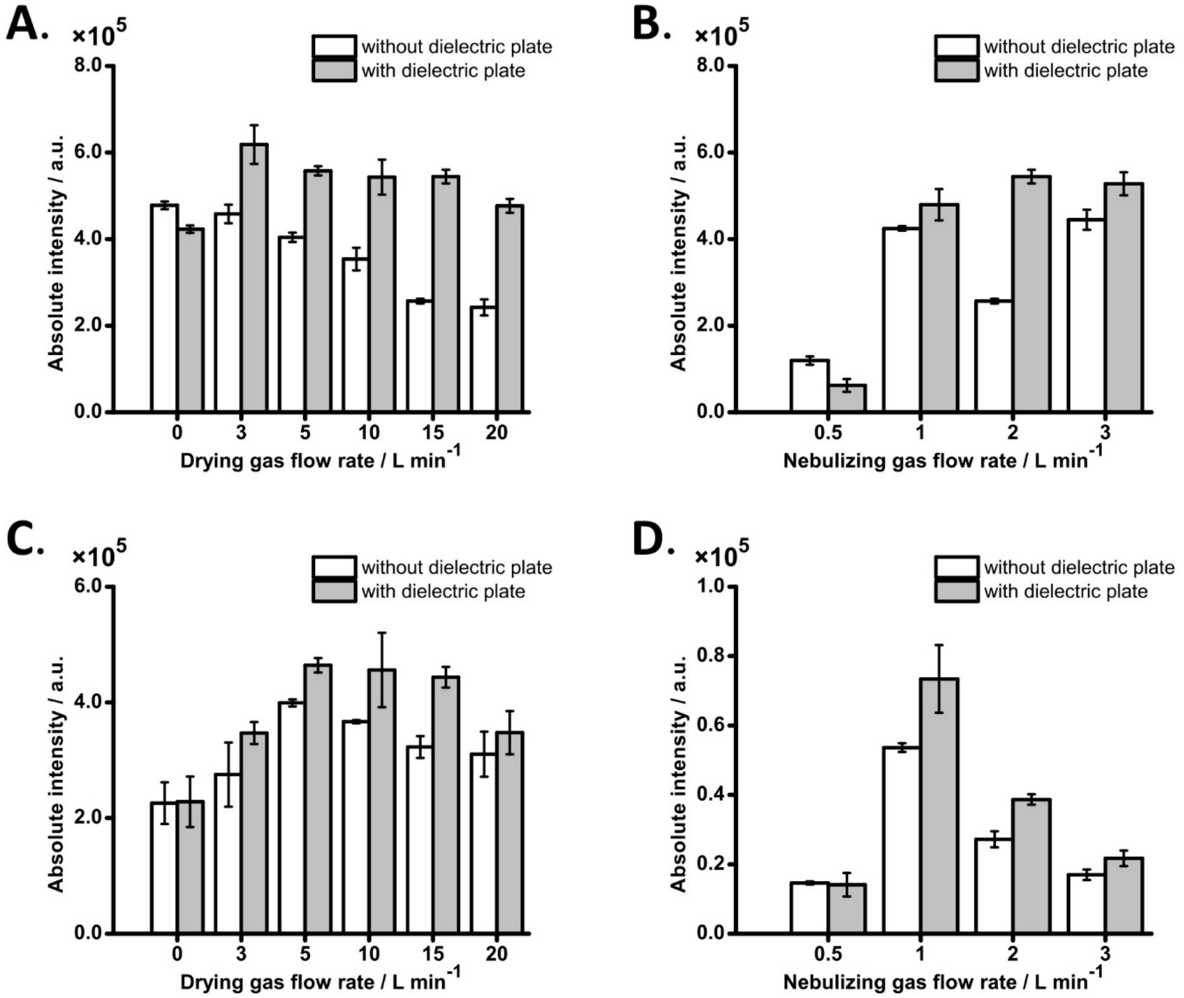

Figure S12. Effect of drying gas flow rate and nebulizing gas flow rate on ion intensities in the presence of dielectric plate. (A,C) Signal intensity vs. drying gas flow rate (nebulizing gas flow rate: 2 L min^{-1}); (B,D) signal intensity vs. nebulizing gas flow rate (drying gas flow rate: 15 L min^{-1}). Horizontal offset between ESI capillary and MS inlet: +1.5 mm. Sample solution: (A,B) 15 µM acetaminophen in 50% (v/v) aqueous ethanol solution (MRM transition: 152→110); (C,D) 15 µM glutathione in 50% (v/v) aqueous ethanol solution (MRM transition: 308→179). Replicates, *n* = 3.

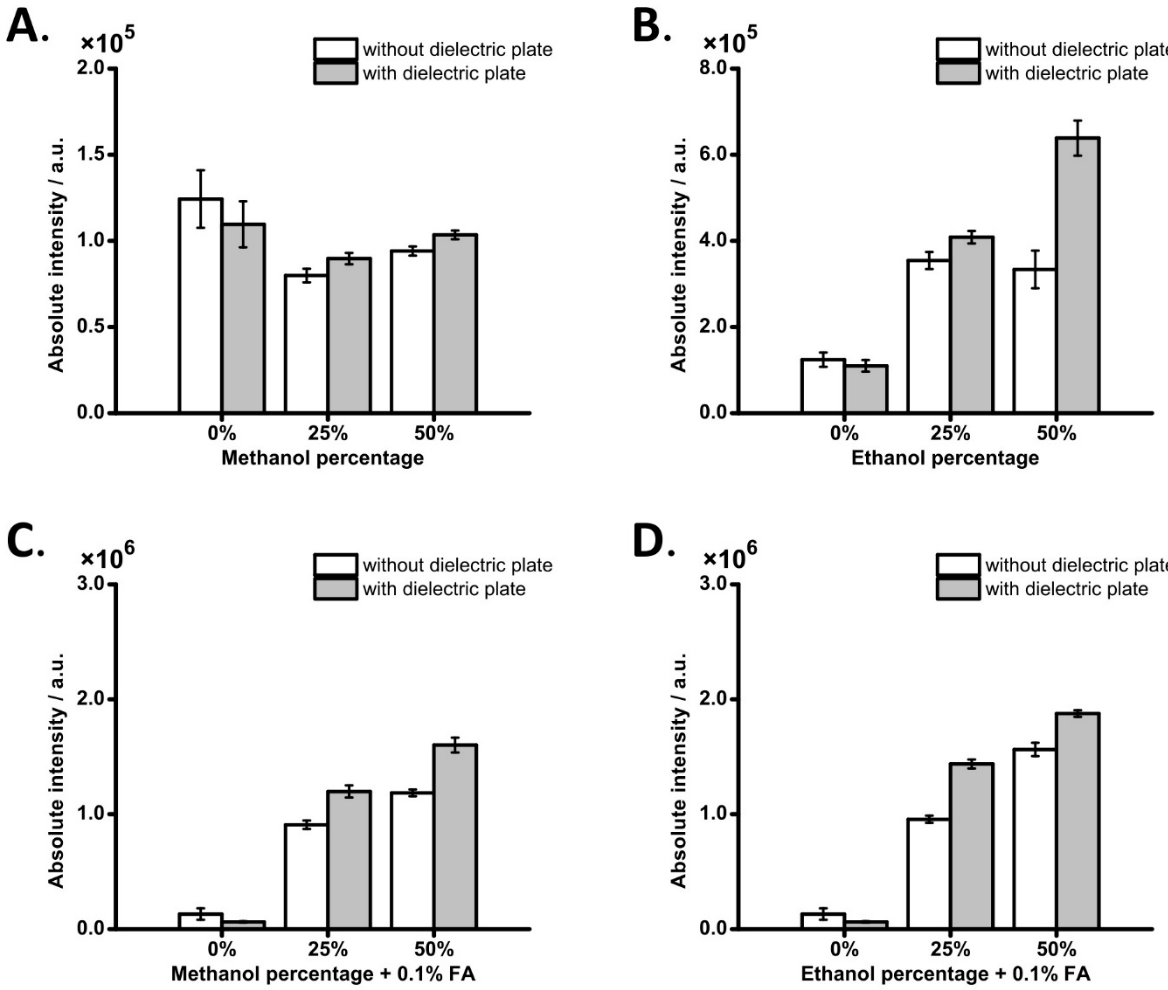

Figure S13. Effect of solvent on ion intensities in the presence of dielectric plate. The signal intensity of different solvent compositions with and without formic acid (FA) was observed: (A) intensities for different percentages (v/v) of methanol without FA; (B) intensities for different percentages (v/v) of ethanol without FA; (C) intensities for different percentages (v/v) of methanol with 0.1% FA; (D) intensities for different percentages (v/v) of ethanol with 0.1% FA. Horizontal offset between ESI capillary and MS inlet: +1.5 mm. Nebulizing gas flow rate: 2 L min^{-1}. Drying gas flow rate: 15 L min^{-1}. Sample: 15 μM acetaminophen (MRM transition: 152→110). Replicates, $n = 3$.

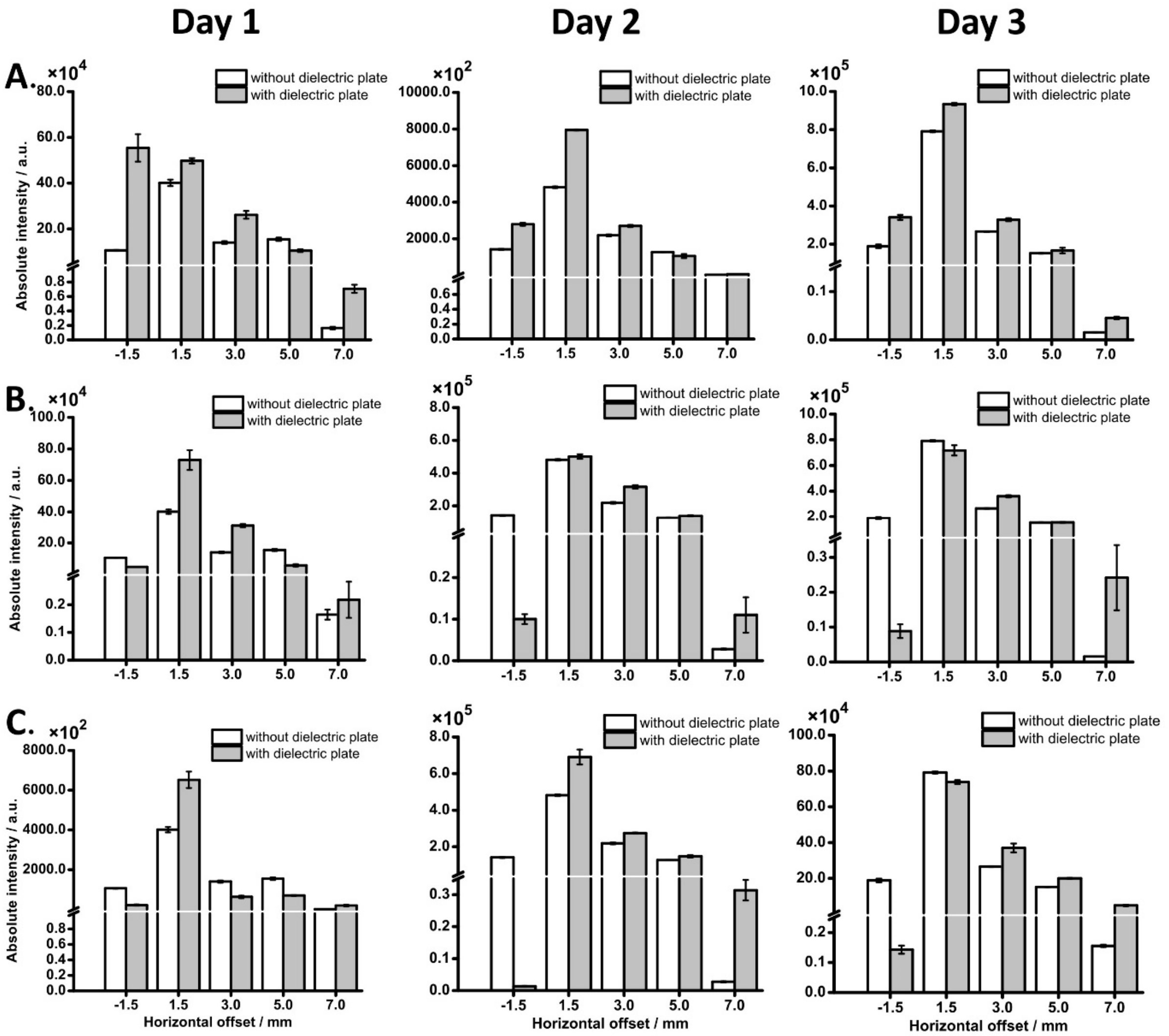

Figure S14. Dependency of signal intensity without and with dielectric plate on distance of electrospray emitter axis from the MS inlet. Three different dielectric plates were tried in this experiment: (A) flat dielectric plate without conical feature; (B) dielectric plate with conical feature; (C) dielectric plate with conical feature and 6 holes for drying gas. Horizontal offset: +1.5 mm. Drying gas flow rate: 15 L min^{-1}. Nebulizing gas flow rate: 2 L min^{-1}. Sample solution: 15 μM acetaminophen in 50% (v/v) aqueous ethanol solution. MRM transition: 152→110. Replicates were done on three different days, while for each day, n = 3.

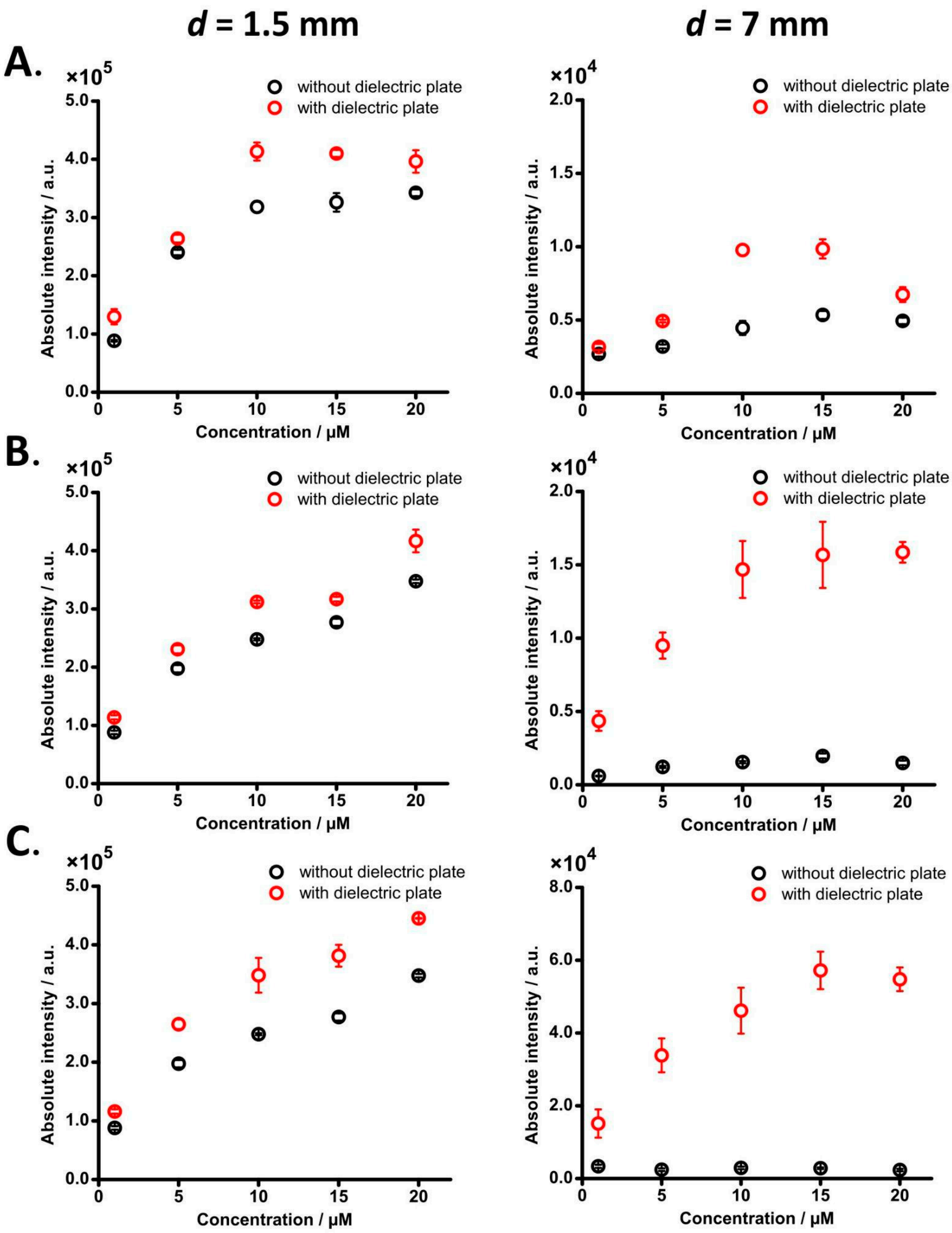

Figure S15. Influence of the analyte concentration on ion intensity at different distances with or without the dielectric plate. Five different concentrations (1 μM, 5 μM, 10 μM, 15 μM, and 20 μM) were analyzed in this experiment in both +1.5 mm (left) and 7 mm (right): (A) Plate A; (B) Plate B; (C) Plate C. Drying gas flow rate: 15 L min^{-1}. Nebulizing gas flow rate: 2 L min^{-1}. Sample solution: acetaminophen in 50% (v/v) aqueous ethanol solution. Sample flow rate: 30 μL min^{-1}. MRM transition: 152→110. Replicates, n = 3.

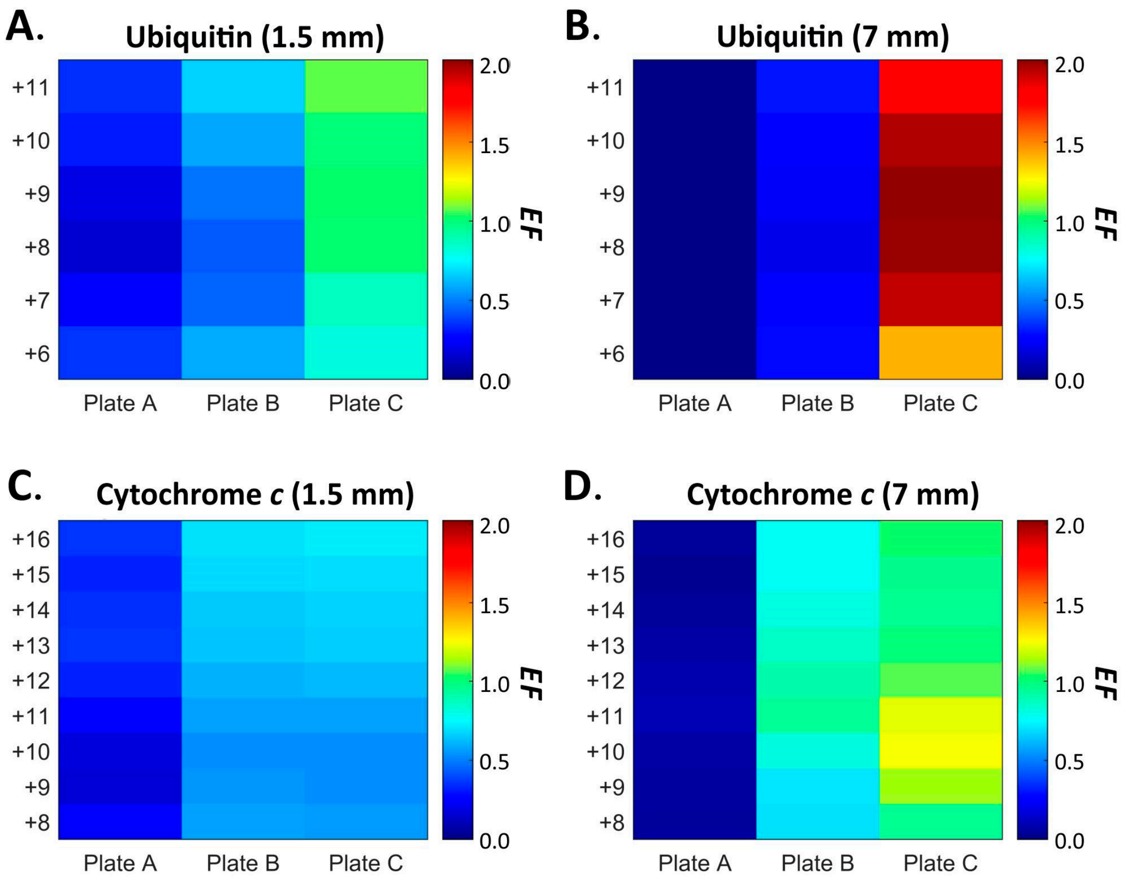

Figure S16. Influence of dielectric plates on signals of different proteins. *EF*s of proteins with different charge states were observed for all dielectric plates in both 1.5 mm and 7 mm: (A) ubiquitin (8600 Da) with horizontal offset of 1.5 mm; (B) ubiquitin (8600 Da) with horizontal offset of 7 mm; (C) cytochrome *c* (12384 Da) with horizontal offset of 1.5 mm; (D) cytochrome *c* (12384 Da) with horizontal offset of 7 mm. Several *m/z* values pertaining to the charge state distribution were followed by single ion monitoring (SIM) in this experiment. Drying gas flow rate: 15 L min^{-1}. Nebulizing gas flow rate: 2 L min^{-1}. Concentration: 15 μM. Solvent: 50% (v/v) aqueous ethanol solution with 10 mM ammonium acetate. Replicates, n = 3.

COMPUTER CODE

Macro for image processing (ImageJ Macro Language)

```
rename("all");
run("Duplicate...", "title=1");
imageCalculator("Subtract create stack", "all","1");#Background subtraction
selectWindow("Result of all");
run("Median...", "radius=2 stack");  #Apply median filter
run("8-bit");            #8-bit conversion
run("Threshold...");     #Open threshold function window
setThreshold(7, 255);    #Default setting of threshold
```
